# Targeting the splicing isoforms of spleen tyrosine kinase affects the viability of colorectal cancer cells

**DOI:** 10.1371/journal.pone.0274390

**Published:** 2022-09-14

**Authors:** Vincent Denis, Nadège Cassagnard, Maguy Del Rio, Emmanuel Cornillot, Nicole Bec, Christian Larroque, Laura Jeanson, Marta Jarlier, Eve Combès, Bruno Robert, Céline Gongora, Pierre Martineau, Piona Dariavach

**Affiliations:** 1 IRCM, Univ Montpellier, Inserm, ICM, Montpellier, France; 2 Institut régional du Cancer de Montpellier (ICM), Montpellier, France; University of Illinois at Chicago, UNITED STATES

## Abstract

Spleen tyrosine kinase (Syk) expression have been both positively and negatively associated with tumorigenesis. Our goal was to evaluate the contribution of Syk and its two splice variants, full length Syk (L) and short isoform Syk (S), in the tumor biology of colorectal cancer cells (CRC). The analysis of Syk expression in primary human colorectal tumors, as well as the analysis of TCGA database, revealed a high Syk mRNA expression score in colorectal cancer tumors, suggesting a tumor promotor role of Syk in CRC. Our analysis showed that Syk (L) isoform is highly expressed in the majority of the tumor tissues and that it remains expressed in tumors in which global Syk expression is downregulated, suggesting the dependence of tumors to Syk (L) isoform. We also identified a small cluster of tumor tissues, which express a high proportion of Syk (S) isoform. This specific cluster is associated with overexpressed genes related to translation and mitochondria, and down regulated genes implicated in the progression of mitosis. For our functional studies, we used short hairpin RNA tools to target the expression of Syk in CRC cells bearing the activating K-Ras (G13D) mutation. Our results showed that while global Syk knock down increases cell proliferation and cell motility, Syk (L) expression silencing affects the viability and induces the apoptosis of the cells, confirming the dependence of cells on Syk (L) isoform for their survival. Finally, we report the promising potential of compound C-13, an original non-enzymatic inhibitor of Syk isolated in our group. *In vitro* studies showed that C-13 exerts cytotoxic effects on Syk-positive CRC cells by inhibiting their proliferation and their motility, and by inducing their apoptosis, while Syk-negative cell lines viability was not affected. Moreover, the oral and intraperitoneal administration of C-13 reduced the tumor growth of CRC DLD-1 cells xenografts in Nude mice *in vivo*.

## Introduction

Spleen tyrosine kinase (Syk) is a 72 kDa non receptor tyrosine kinase that contains two tandem Src homology 2 domains at the NH2 terminus and a kinase domain at the COOH terminus. Syk has two alternatively splice isoforms: full length Syk (L) and short form Syk (S) that lacks a 69-nucleotide exon. Syk (L) is present in both the cytoplasm and the nucleus of the cells due to a nuclear localization signal present in the 23 residues of interdomain B encoded by exon 9, missing in Syk (S). Consequently, Syk (S) is located exclusively in the cytoplasm of the cells.

Syk is widely expressed in hematopoietic cells where it plays a key role in the activation of the cells following the stimulation of antigen and Fc Receptors. Following receptor engagement and clustering, Syk is recruited to the receptor through the binding of its SH2 domains to a double phosphorylated immunoreceptor tyrosine-based activation motif or ITAM. This activates Syk through auto- and trans-tyrosine phosphorylation. Activated Syk then catalyzes the phosphorylation of protein substrates primarily on tyrosines, however Syk also has the ability to phosphorylate some proteins on serine. These phosphorylations lead to the activation of signaling cascades that include the PI3K/Akt, Ras/Erk, PLCγ/NFAT, and IKK/NFκB pathways. The critical involvement of Syk in the activation of immune cells has made it a popular target for anti-inflammatory therapeutics directed against diseases such as allergic asthma, rheumatoid arthritis, lupus erythematosus and thrombocytopenic purpura (reviewed in REFS [1,2]).

Syk plays also an important role in epithelial solid cancers where its expression is a marker predicting either poor or favorable outcome [3]. Indeed, the contribution of Syk in tumor biology depends on cancer types and it can be of three origins: 1) pro-survival role by stabilizing anti-apoptotic proteins (MCL-1/BCL-2 family) [4,5] and by suppressing c-JUN expression [6]. This activity underlies many of the tumor promoting activities of the kinase; 2) negative regulator of EMT by enhancing cell-cell and cell-matrix adhesion [7,8]. This activity underlies many of the tumor suppressive activities of the kinase; and 3) regulator of mitotic progression through its centrosomal kinase activity associated with γ-tubulin [9] and by controlling the cell cycle G2-M phase progression [10].

Many studies have investigated the overall expression of Syk as well as the expression of the splicing variants of Syk and their contribution to tumorigenesis. Coopman and co-workers were the first group reporting the role of Syk as a suppressor of tumorogenesis in breast cancer cells [11]. In this model, the loss of Syk expression is due to the hypermethylation of Syk gene promoter, and it is frequently observed in primary breast tumors, while the unmethylated promoter is found in adjacent normal breast tissue [12]. The loss of Syk expression is also found in gastric cancer [13], hepatocellular carcinoma (HCC) [14] and melanoma [15].

On the opposite, the role of Syk as an oncogene is frequently reported in squamous carcinomas of head and neck (SCCHN) [16] where its expression enhances cell migration. Interestingly, high expression of Syk is significantly associated with recurrence and shorter survival in SCCHN patients. In ovarian cancer, the expression of Syk increases with tumor grade, and the silencing of Syk expression inhibits anchorage-independent growth and induces apoptosis in ovarian cancer cells [6]. A similar pro-survival role for Syk is seen in retinoblastoma cells, where both the treatment with Syk inhibitors and knockdown of Syk expression result in a dramatic increase in apoptosis of retinoblastoma cells [17].

Accumulating evidences suggest an active but opposing role of Syk (L) and Syk (S) isoforms on the growth properties of cancer cells, possibly due to different biologic functions for the two isoforms. Wang and co-workers [18] showed that in primary breast tumors, Syk (L) was present in both tumor and matched normal mammary gland, and that ectopic expression of Syk (L) in a cellular model suppressed cell invasiveness. They also showed that Syk (S) was expressed in cancerous tissues and not in normal mammary gland, and Syk (S) ectopic expression did not reduce cell invasiveness *in vitro*. On the opposite, Prinos et al [6] reported that changing the Syk alternative splicing pattern by decreasing Syk (L) and increasing Syk (S) expression in an ovarian cancer cell line induced apoptosis and altered cell survival and mitotic progression. A differential expression pattern of Syk was also found in HCC where Syk (L) mRNA expression was downregulated in 38% of the tumor samples while Syk (S) mRNA expression was detectable in 40% of the tumor samples and none in the normal liver tissue samples [19]. Moreover, patients with a Syk (S)-positive HCC had a worse overall survival compared to patients with a Syk (S)-negative HCC.

Little is known on the contribution of Syk and its splicing variants in the tumor biology of colorectal cancer. Yang et al. [20] showed a loss of overall Syk mRNA expression in half of CRC patients due to the hypermethylation of the Syk promoter region, which was associated with a higher tumor stage and reduced five-year overall survival in a heterogeneous group of stage I-IV colon and rectum carcinoma. Ni et al. [21] investigated the expression levels of the splice variants of Syk in 26 pairs of CRC and adjacent non-cancerous tissues. Their study showed that while Syk (L) is downregulated in 69% of tumor tissue samples compared to the adjacent non-cancerous tissue, the expression of Syk (S) remains stable, suggesting that Syk (L) but not Syk (S) is associated with tumor suppressing activities. Coebergh et al. [22] studied the prognostic role of Syk in a cohort of 160 chemonaive lymph node negative colon cancer patients, and they found that Syk (S) high mRNA expression correlates with metastatic relapse with hepatic lesions. However, they did not observe any association between mRNA expression of the splice variants and tumor grade, nor any evidence supporting a tumor suppressor role for Syk (L).

Our goal was to evaluate the role of Syk alternative spliced isoforms in cancer biology of colorectal carcinomas. For this purpose, we used two approaches: i) short hairpin RNA (shRNA) tools to target the global expression of Syk as well as its alternative splicing variant Syk (L); ii) the use of compound C-13, an original non-enzymatic inhibitor of Syk isolated in our group [23]. Our functional studies demonstrated that CRC cell lines depend on Syk long isoform for their survival, since Syk (L) expression silencing affected the viability and induced the apoptosis of the cells. Moreover, the application of compound C-13 phenocopied Syk (L) expression silencing *in vitro*, and its oral and intraperitoneal administration reduced the tumor growth of CRC DLD-1 cells xenografts in Nude mice *in vivo*.

## Materials and methods

### Drugs and reagents

Syk non-enzymatic inhibitor C-13 was purchased from ChemBridge, Inc (San Diego, CA) (ID number 6197026) and was dissolved to a final concentration of 10 mM in dimethylformamide (DMF). Syk catalytic inhibitor R406 was from Santa Cruz Biotechnology and was dissolved to a final concentration of 10 mM in dimethyl sulfoxide (DMSO). Syk inhibitor Piceatannol was from Sigma-Aldrich and was dissolved to a final concentration of 40 mM in DMSO. Recombinant human EGF (1 mg/mL) was from Sigma-Aldrich. Cetuximab (ERBITUX^®^) (5 mg/mL) was purchased from Merck (Darmstadt, Germany). All stock solutions were aliquoted and stored at -20C. All reagents unless otherwise mentioned were from Sigma (St Louis, Mo).

### Cell culture

All cell lines were from the ATCC and were cultured in antibiotic- and antimycotic-free medium (Gibco). The human colorectal adenocarcinoma HCT-116, HT29, SW480 and SW620 were grown in RPMI Glutamax medium, and DLD-1 cell line was grown in DMEM/F12 (50/50) Glutamax medium. All culture media were supplemented with 10% (v/v) FBS. The DIFI cell line was grown in RPMI Glutamax medium supplemented with 20% (v/v) FBS.

For EGF induction experiments, cells were serum-starved for 12 hours, and they were subsequently treated with the recombinant human EGF (50 ng/mL) or with EGF (50 ng/mL) + Cetuximab (50 μg/mL) in serum free culture medium, for 10 minutes.

Cell propagation and passaging were as recommended by the American Type Culture Collection (ATCC). For cell enumeration, cells were detached with TrypLE Express Enzyme (Gibco), and they were counted using Z1 Particle Counter (Beckman Coulter). The cell lines were tested and authenticated by short-tandem repeat profiling (LGC Standards and Eurofins Genomics). All experiments were performed at least three times.

### Syk silencing by small hairpin RNA

To design long isoform-specific and global Syk specific shRNA, we used siRNA sequences from Pinos et al. [6]. Four different shRNA expressing pSIREN vectors with the puromycin selection marker and specific to Syk were used. shRNA G1, G2 inhibit global gene expression and shRNA L1, L2 are alternative exon-specific. shRNA LUC with Luciferase sequence was used as negative control. Lentiviral production was performed by cotransfecting 1x10^6^ 293T cells (for lentiviral packaging) with 3 μg of pSiren vector in which anti-Syk shRNAs where cloned, 1 μg of the packaging vector gagpol, and 1 μg of envelope vector, using JetPRIME (Polyplus), according to the manufacturer’s instructions. Lentiviral particles were harvested, filtered and then used to infect 1x10^6^ cells for 48 hours. Following shRNA transduction, cells were selected with 1 *μ*g/mL puromycin for one week and stable clones were pooled.

### shRNA target sequences

**shSykG1**: 5’ GCAGATGGTTTGTTAAGAG 3’; **shSykG2**: 5’ GTCGAGCATTATTCTTATA 3’; **shSykL1**: 5’ GTTCCCATCCTGCGACTTG 3’; **shSykL2:** 5’ GGTCAGCGGGTGGAATAAT 3’; **shLUC:** 5’ TTACGCTGAGTACTTCGA 3’.

### RNA extraction and quantitative PCR

Total RNA extractions were performed from 0.5 to 1.10^6^ cells using the ZR RNA MiniPrep Kit (Zymo Research) as recommended by the manufacturer. Reverse transcription was performed on 1 μg RNA using PrimeScript Reverse Transcriptase (Takara Bio.), SYBR Premix Ex Taq 2X master mix (Takara Bio.) and 5 pmol of both forward and reverse primers. Quantitative PCR reactions were performed on 1 ng cDNA. HPRT was amplified as internal control.

### qPCR primer sequences

**Syk L**: for 5’ TCAGCGGGTGGAATAATCTC 3’; rev 5’ TGCAAGTTCTGGCTCATACG 3’
**Syk S**: for 5’ TGGCAGCTAGTCGAGCATTA 3’; rev 5’ CAGGGGAGGACGCAGGAT 3’
**Syk L+ S**: for 5’ GAAGCCATATCGAGGGATGA 3’; rev 5’ CCACATCGTATGTCCAGCAC 3’
**HPRT**: for 5’ CTGACCTGCTGGATTACA 3’; rev 5’ GCGACCTTGACCATCTTT 3’

### Cell growth inhibition assay (2D assay)

Cell growth was evaluated using the CellTiter-Glo^®^ Luminescent Cell Viability Assay (Promega) according to manufacturer’s recommendations. Briefly, 3,000 cells/well were seeded in 96-well plates. After 24 hours, drugs were added in serial dilution. Cells were incubated for 96 hours, after which, a volume of CellTiter-Glo^®^ reagent equal to the volume of cell culture medium was added to each well. The plate was shaked on an orbital shaker for 15 minutes at room temperature to induce cell lysis. The luminescence was recorded using a Thermo Fisher Scientific Multiskan EX plate reader. The IC50 was determined graphically from the cell growth curves.

### Migration

DLD-1 or HCT-116 cells were serum starved for 16 h in a 1%-FBS-medium. Cells were detached with TrypLE Express Enzyme (Gibco). 5x10^4^ cells were resuspended in a 1%-FBS-medium and they were seeded on Fluoroblok 24-well plate permeable inserts, with 8 μm pores (Corning). Following a 20-22h incubation with chemoattractant (10% FBS in RPMI medium), the inserts were transferred in a 2^nd^ 24-well plate containing a 4 μg/mL Calcein AM solution (Sigma Aldrich). The fluorescence was read on an inverted fluorescence microscopy and migrated cells were counted with ImageJ software.

### Invasion

Same as migration, but the Fluoroblok insert was coated with 100 μL of 300 μg/μL BD Matrigel Matrix (BD Biosciences).

### Apoptosis assay

A total of 2.5x10^5^ cells were plated, and after 24 hours, they were incubated with the indicated concentrations of compound C-13 for 2 hours. ShRNA-tranduced cells were plated following one week puromycin selection, and grown for 48 hours. Cells were stained with APC-labeled Annexin V and 7-aminoactinomycin D (Biolegend). For apoptosis determination, Annexin V- and 7-ADD-positive cells were quantified using a Gallios Cytometer (Beckman Coulter) and the Kaluza Software (Beckman Coulter).

### Cell-cycle analysis

To determine the cell-cycle distribution, 2.5x10^5^ cells were plated in 25 cm2 flasks. After 48 hours, cells were synchronized by serum starvation during 12 hours. Quiescent cells were induced to re-enter the cell cycle by serum refeeding. After 15 hours, cells were washed in ice-cold PBS, fixed in 75% ethanol, and labeled with 40 mg/mL propidium iodide (Sigma-Aldrich) containing 100 mg/mL RNase A (Sigma). Cell-cycle progression was analyzed by flow cytometry with a Gallios Cytometer (Beckman Coulter) and quantified using the Kaluza Software (Beckman Coulter).

### Immunofluorescence

5x10^4^ cells per well were seeded on Lab-Tek II glass chambers (Thermo Fischer Scientific). After 24 hours, cells were washed with ice-cold PBS, and they were fixed and permeabilized with ice-cold methanol (Sigma Aldrich) for 15 minutes at -20°C. Cells were incubated in PBS containing 2% BSA for 30 minutes at room temperature. Cells were incubated with the primary and the fluorescent antibodies for 1h at room temperature. The primary antibodies used were: rabbit polyclonal Syk (1:100) (sc-1077, Santa Cruz Biotechnology); mouse monoclonal anti-beta Tubulin (1:200) (T4026, Sigma). Primary antibodies were detected by Alexa Fluor 488-conjugated anti-mouse IgG (1:400) (715-546-151, Jackson ImmunoResearch laboratories) and APC-conjugated anti-rabbit IgG (1:200) (711-136-152, Jackson ImmunoResearch laboratories) antibodies, respectively. Nuclei were stained with Hoechst 33342 dye. After washing, the slides were mounted in VECTASHIELD Mounting medium (H-1400, Vector laboratories) and were analyzed visually using Zeiss Imager M2 microscope.

### Patients and ethics statement

Colorectal cancer patients with synchronous and unresectable liver metastases were enrolled in a prospective study at the ICM Cancer center from January 2000 to June 2004 [24]. Normal colon, colon cancer and hepatic metastasis samples were collected at the time of surgery, prior to chemotherapy. The study was approved by ICM (Institut du Cancer de Montpellier) CORT (Comité de Recherche Translationnelle) ethical committee and all participating patients were informed of the study and had to provide signed written informed consent before enrollment. The ethics approval to use those cancer samples was provided by the ICM/CORT IRB committee, which collected the consent of all patients. All experimental protocols using these human samples were carried out in accordance with the French Guidelines and Regulations for Human Samples.

### Tissue samples

From 13 CRC patients mentioned above, we collected pairs of colon primary tumors (CT) and normal colon mucosas (CN). These 13 pairs of samples were used to study the protein expression pattern of Syk expressed in CT versus the corresponding CN. Prior to western blot analysis, tumor tissue samples from patients were directly grinded in lysis buffer (150mM NaCl, 10mM Tris pH 7.4, 1mM EDTA, 1mM EGTA, 1% SDS, 1% Triton X-100, 0.5% NP-40, 2mM PMSF, 100mM NaF, 10mM sodium orthovanadate, one cocktail protease inhibitor tablet for 10 mL) using a Mixer Mill^®^ MM 300 unit (Qiagen, Valencia, CA). Protein concentration was determined with the Bradford assay (Pierce Coomassie Plus Protein Assay). Then, 50 μg of total proteins solubilized in 1x Laemmli sample buffer (10% glycerol, 5% β-mercaptoethanol, 2.3% SDS, 62.5 mM Tris-HCl (pH 6.8), and 0.1% bromophenol blue) were resolved by 10% SDS-PAGE.

### Cell culture samples

Cells were solubilized in DOC modified lysis buffer (1% Igepal, 0.25% sodium deoxycholate, 0.1% SDS in PBS buffer supplemented with protease and phosphatase inhibitors) and the protein concentration was determined (PIERCE BCA Protein Assay, Thermo Fisher Scientific). Equivalent total protein amounts (50 μg per sample) solubilized in 1x Laemmli sample buffer were subjected to 8–10% SDS-PAGE.

### Immunoblotting

Following electrotransfer to Amersham nitrocellulose membrane (GE Healthcare, pore size 0.45μm) using a standard protocol, the membranes were first stained with Ponceau S (Sigma), to confirm protein loading equivalence, then blocked 1 hour at room temperature with gentle shaking in TBS containing 0.1% Tween-20 (TBST) and 5% skimmed milk. Primary antibodies were diluted in blocking solution and hybridized overnight at 4°C with gentle shaking. The antibodies used were: mouse monoclonal anti-Syk (1:500) (4D10, #sc1240 Santa Cruz Biotechnology); rabbit polyclonal anti-Phospho-Akt (1:2,000) (#4060, Cell Signaling Technology); mouse monoclonal anti-Phospho-p44/42 MAP Kinase (1:2,000) (#9106, Cell Signaling Technology); rabbit polyclonal anti-p44/42 MAP Kinase (1:1,000) (#9102, Cell Signaling Technology); mouse monoclonal anti-beta Tubulin (1:5,000) (SAB4200715, Sigma); rabbit polyclonal anti-EGF Receptor (1:1,000) (#2232, Cell Signaling Technology); goat polyclonal anti-Akt (1:200) (sc-1618, Santa Cruz Biotechnology); HRP-linked anti-goat antibody (1:5,000) (sc-2033, Santa Cruz Biotechnology); HRP-linked anti-rabbit antibody (1:5,000) (sc-2004, Santa Cruz Biotechnology); HRP-linked anti-mouse antibody (1:5,000) (sc-2314, Santa Cruz Biotechnology).

Membranes were washed 5 times (10 min. each) in TBST and appropriate HRP-conjugated secondary antibodies were diluted in blocking solution and hybridized 1 hour at room temperature with gentle shaking. After 5 washes in TBST, the proteins were detected by chemiluminescence generated with Western Lightning Chemiluminescence Reagent Plus (GE Healthcare) and detected on PXi Syngene Chemiluminscence system. For reprobing, membranes were incubated at room temperature for 10 min in stripping solution (100 mM Glycine pH 2.8; 0.1% Igepal and 1% SDS) with gentle shaking. Membranes were then washed (3 times, 5 min. each) with TBST and blocked as described previously, before rehybridization.

### Mass spectrometry

#### Syk pull-down assays

DLD-1 cells were serum starved, then separated in two batches. One batch was incubated with EGF (50 ng/mL) for 10 minutes at 37°C. Both batches of EGF-untreated and EGF-treated cells were washed with ice-cold PBS containing phosphatase inhibitors (100 mM sodium fluoride, 5 mM sodium orthovanadate), and cells were lysed for 15 minutes on ice with lysis buffer (PBS supplemented with 0.5% sodium deoxycholate, 1% NP-40, 0.1% SDS). Cell lysates were clarified by centrifugation for 15 minutes at 4°C at 16,000 g. The total protein content of the soluble fraction was quantified using the BCA assay kit (Interchim, France).

5.5 mg of protein lysates from serum-starved and EGF-treated DLD-1 cells were incubated with agarose-conjugated anti-Syk 4D10 monoclonal antibody (#sc-1240, Santa Cruz Biotechnology) and agarose-conjugated mouse IgG (#sc-2343, Santa Cruz Biotechnology, control) for 2 hours at 4°C. Beads were washed 3 times in lysis buffer, then they were resuspended in 1x Laemmli sample buffer and the protein contents were analyzed by SDS-PAGE.

#### Sample preparation

Proteins from the immunoprecipitation batches were resolved by 8% SDS-PAGE and detected with Coomassie-brilliant blue staining. Lanes were cut into five fractions. Gel pieces were subjected to in-gel alkylation and digestion with trypsin.

#### LC separation and MS/MS detection

Desalted peptide mixtures were fractionated on an Ekspert 425 nanoLC system (Eksigent) equipped with a C18 column (Discovery BIO Wide Pore, Supelco). The mobile phases were solvent A (water, 0.1% FA) and B (acetonitrile, 0.1% FA). Injection was performed with 98% solvent A at a flow rate of 5 μl/min. Peptides were separated at 30◦C with the following gradient: 2% to 40% B for 105 min, 40–80% B for 5 min. The column was washed with 80% solvent B for 5 min and equilibrated with 98% solvent A. Peptide separation was monitored online with the coupled TripleTOF 5600 mass spectrometer (Sciex). The total ion chromatogram acquisition was made in information-dependent acquisition (IDA) mode using the Analyst TF v.1.7 software (Sciex). Positive ion profiling was performed from m/z 350–1,500, followed by a MS/MS product ion scan from m/z 100–1,500 with the abundance threshold set at more than 100 cps. The accumulation time for ions was set at 250 ms for MS scans, and 100 ms for MS/MS scans. Target ions were excluded from the scan for 10s after detection. The IDA advanced “rolling collision energy (CE)” option was employed to automatically ramp up the CE value in the collision cell as the m/z value was increased. A maximum of 25 spectra were collected from candidate ions per cycle.

#### Data analysis

Combining the 5 runs per sample, peptide and protein identifications were performed in Uniprot/Swiss- Prot2016_01 database by ProteinPilotTMSoftware V 4.5 (Sciex). This software calculates a confidence percentage that reflects the probability that the hit is a false positive, meaning that at 99% confidence level (unused score>2), there is a false positive identification chance of about 1%.

#### Bioinformatic analysis

TCGA gene and protein expression matrix were recovered for the COAD and READ cohorts using API GDC portal with curl function. They were merged together to generate the CRC (colorectal) dataset based on 622 individuals. About 18 thousands genes were expressed in more than 5% of the samples. Only 52 patients had RNAseq data for the tumor and the normal tissue. Fifty tumor samples had both mRNA and protein expression values. Splicing event concerning the exon 9 of Syk were recovered from TCGA SpliceSeq database as PSI value from the web interface [25]. PSI values were obtained for all samples recovers from TCGA. We assigned NA PSI value to zero in the present analysis. Tumor samples were separated based on Syk exon 9 PSI value between low (<0.25), medium and high (>0.75). Differential gene expression among tumor samples was calculated using edgeR. We kept genes that were differentially expressed between Low and High PSI tumors. Analysis was performed on the 10,000 most variable genes. Variation was calculated on the log10 of gene expression values. Graphs were generated using R version 3.6.2 and ggplot2 library. Complex heatmap library was used to draw heatmaps. Clinical data were recovered from integrated Pan-cancer resource published by Liu et al. [26]. Three CRC samples had no clinical information. MSI features were obtained from Bonneville et al. [27]. Information was missing for 43 patients. K-Ras status of CRC patients was downloaded at cbioportal. CMS labels were matched using CMS subtyping calls: (https://github.com/Sage-Bionetworks/crc-cms-kras/blob/master/020717/cms_labels_public_all.txt).

### *In vivo* studies

#### Xenografts

1x10^6^ DLD-1 tumor cells were injected subcutaneously in the left flank of 6-week-old female athymic nu/nu mice (Charles River Laboratories). Tumors were detected by palpation and measured with a caliper three times per week. Mice were euthanized by cervical dislocation when the tumor volume reached 1.500 mm^3^.

During the experiments, health monitoring of mice was performed to ensure their health, including monitoring animals from external sources as well as animals kept in the experimental unit; housing and hygienic monitoring, pathogen detection, diagnostic measures to enable disease control and to maintain the health status of mice. Among the efforts to alleviate suffering, we adapted the protocol to minimize distress for the mice. Consequently, we defined tumor growth (a maximum size of 1500 mm3) instead of survival endpoint. We also refined the sample collection methods and instituted species-specific husbandry refinements in order to attenuate anxiety and stress. The number of animals used was reduced to the absolute minimum necessary, based on appropriate statistical sample size determination.

Ethical approvals were obtained by the local ethics committee (Ethics Committee approved by the French Ministry, animal facility approval D34-172-27, and protocol approval CEEA-LR-10830 / 2018032611365978#10830).

#### Tumor treatment

When tumors reached the volume of 40–70 mm3, mice were randomized in groups of 7 to 10 animals, and they were treated three times per week for 3 weeks with: (i) 0.2 mL of 0.9% sodium chloride solution by intraperitoneal injection of 0.35 mg/kg C-13; (ii) 0.2 mL of 0.9% sodium chloride solution by intraperitoneal injection of DMF (vehicle, control group). (iii) 100 mg/kg C-13 dissolved in 0.1% carboxymethylcellulose solution by gavage; (iv) DMF dissolved in 0.1% carboxymethylcellulose solution (vehicle; gavage control group). Mice were euthanized when the tumor volume reached 1500 mm3.

#### Statistical analysis

For *in vitro* experiments, data were compared using the unpaired Student’s t test. For *in vivo* experiments, data were described using median, mean and standard deviation. Linear mixed regression models were used to determine the relationship between tumor growth and number of days after implantation. The variables included in the fixed part of the model were the number of days after implantation and the treatment group; their interaction were also evaluated. Random intercepts and random slopes were considered for time effect. The model coefficients were estimated by maximum likelihood. Statistical significance was set at the 0.05 level. Statistical analyses were conducted using the STATA 16.0 software (StataCorp, TX, USA).

## Results

### Syk isoforms expression in CRC cell lines

**Fig 1:**
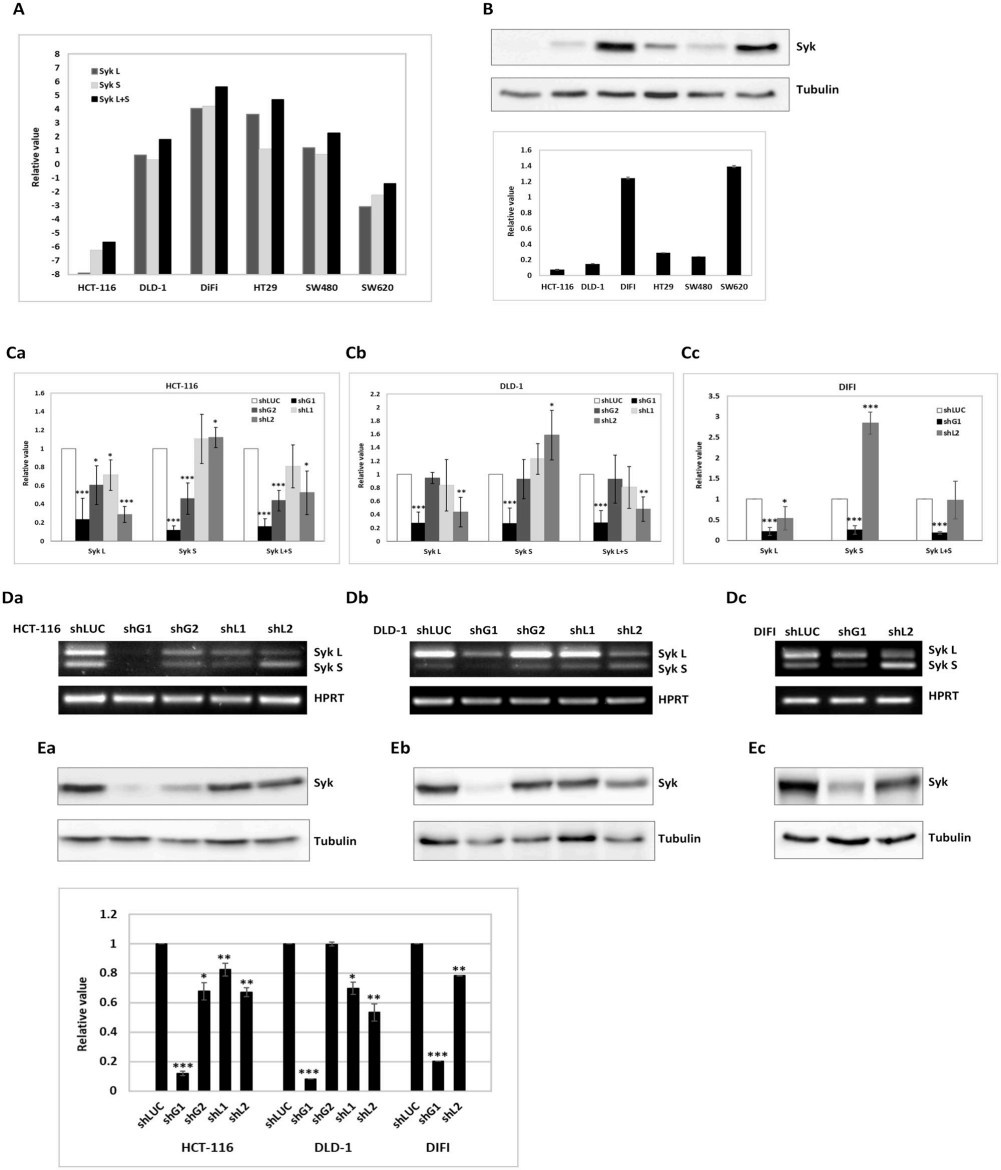
Targeting of Syk alternative spliced isoforms. (A) The relative expression of Syk (L), Syk (S) and total Syk (L+S) in colorectal carcinoma cell lines was detected by qPCR; HPRT was used as an internal control. The Y-axis of the plot is expressed in a base-2 log scale. The figure is representative of three independent experiments. (B) *Top panel*- The expression of total Syk was evaluated by western blot. *Bottom panel*- Densitometric quantification of immunoblot analyses of Syk expression over control. The impact of shRNAs targeting the pre-mRNAs encoding Syk proteins evaluated by: (C) RNA expression monitored by qPCR, (D) RT-PCR; HPRT was used as an internal control, and (E) western blot, and compared to mock transduced (shLUC) HCT-116 (panel Ea), DLD-1 (panel Eb) and DIFI (panel Ec) CRC cells. Experiments were performed in three biological and three technical replicates, and an average for each cellular phenotype was calculated for each shRNA. (E) *Bottom panel*- Densitometric quantification of Syk expression over control, for immunoblots shown in panels Ea, Eb and Ec. shRNA G1, G2 inhibit global gene expression & shRNA L1, L2 are alternative exon-specific. shRNA LUC with Luciferase sequence was used as negative control. The qPCR relative expression values for the overall gene expression Syk (L+S), long splice isoform (Syk L) and short splice isoform (Syk S) were normalized against HPRT used as control. Error bars represent the mean ± SD of three independent experiments (*, P<0.05; **, P<0.01; ***, P<0.001, compared with control). For the western blots, total proteins were extracted one week post-transfection and equal amount of proteins were loaded on an 8% SDS-PAGE. The anti-Syk and anti-Tubulin antibodies used are described in materials & methods. Tubulin served as a loading control.

We assessed the expression levels of total Syk (L+S) as well as Syk (L) and Syk (S) isoforms in six CRC cell lines by RT-qPCR (Fig 1A). This analysis showed that these cell lines express different levels and splicing isoform ratios of Syk: the expression levels of global Syk (L+S) and that of Syk long isoform transcript are 5 to 4000 fold higher in HT29 and DIFI cell lines compared to the four other cell lines. Among the six cell lines, DIFI expresses the higher level of Syk short isoform transcript, which is 8 to 1800 fold higher than the other cell lines. The mRNA level is well correlated with the protein expression level detected by western blot analysis (Fig 1B), except for SW620 that expressed a high level of protein despite a low level of mRNA, suggesting that unknown post-translational mechanisms may affect Syk expression. However, we could not visualize at the protein level, the two Syk isoforms due to their co-migration by SDS-PAGE and the absence of suitable isoform-specific antibodies.

### Syk long isoform is implicated in CRC cell lines survival and mitosis

Our goal was to evaluate the role of Syk alternative spliced isoforms in cancer biology of colorectal carcinomas. Short hairpin RNA (shRNA) and RNA interference (RNAi) have become preferred tools for evaluating the impact of gene expression on the viability of cancer cells, and for understanding individual isoform functions. Compared to RNAi, shRNA can be integrated into genomic DNA for long-term or stable expression, leading to a longer knockdown of the target mRNA and a durable down-regulation of protein expression, thus allowing the evaluation of protein knockdown effects on cellular functions.

For our purpose, we used shRNA-based isoform-specific silencing to investigate the effects of Syk splicing modulation on cell growth and cell survival of HCT-116, DLD-1 and DIFI colorectal cell lines. We compared the effects of shRNA on global and long-isoform specific expression by RT-qPCR and RT-PCR (Fig 1C and 1D). Global shRNAs (shG1, shG2) equally inhibited the expression of long and short splice isoforms of Syk. Long Isoform specific shRNAs (shL1, shL2) inhibited the accumulation of the long splice isoform while proportionally reducing the overall gene expression level. Interestingly, Syk (L) depletion increased the expression of the short isoform to 1.6 fold in DLD-1 and to 2.8 fold in DIFI cell line. These observations were confirmed by western blot analysis using antibodies against total Syk protein to evaluate the impact of the shRNA-induced mRNA downregulation at the protein level (Fig 1E). Our results showed that among the global shRNAs, shG1 and among the isoform specific shRNAS, shL2 were the most efficient ones for reducing Syk gene expression both at the mRNA and at the protein levels.

**Fig 2:**
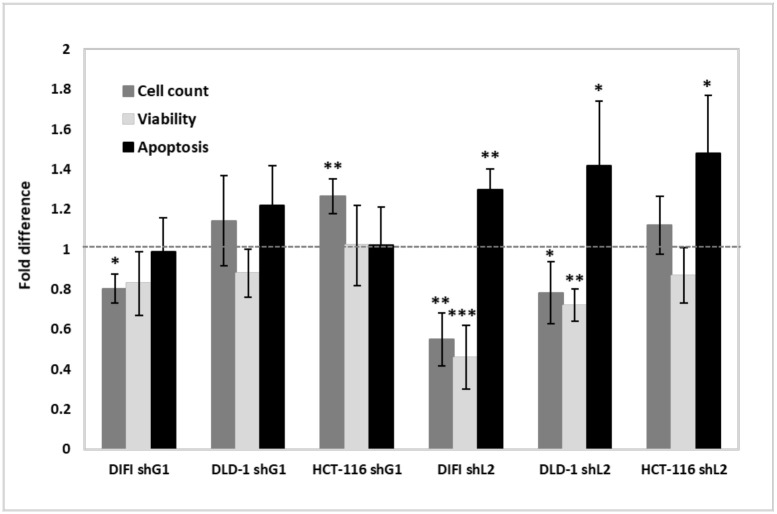
Changing the splicing pattern of Syk affects cell viability and induces apoptosis. The impact of shRNAs targeting global Syk (shG1) & long-isoform-specific Syk (shL2) pre-mRNAs on cellular phenotype evaluated by: cell proliferation, viability and apoptosis, and normalized to unrelated-transduced shRNA (shLUC) HCT-116, DLD-1 and DIFI CRC cells. Cell numbers were monitored using cell counts (Countess II, Promega), apoptosis was monitored using Annexin V-FITC marker and analyzed by flow cytometry (Gallios Beckman Coulter) and viability was monitored using CellTiter-Glo Luminescent cell viability assay (Promega). The relative changes were calculated and used to generate the bar graphs. The error bars represent the variation observed in three biological and three technical replicate (*, P<0.05; **, P<0.01; ***, P<0.001, compared with control).

Syk has been previously described as implicated in cell-survival pathways and apoptosis. We evaluated the impact of shRNA-based global and long-isoform specific silencing of Syk on the viability of CRC cells. Inhibiting the expression of the long isoform of Syk resulted in an increase of apoptotic marker-positive cells in the three cell lines (Fig 2). Accordingly, cell proliferation and the ratio of viable cells were markedly decreased by shRNA-mediated silencing of the long isoform of Syk (Fig 2). On the opposite, Syk general knock down with global shRNA shG1 did not significantly affect cell apoptosis and viability. However, it increased cell proliferation of HCT-116 and DLD-1 cells, and had the opposite effect in DIFI cells where Syk general knock down reduced cell proliferation (Fig 2). Transfection of an unrelated shRNA (shLUC) did not affect any of these cellular phenotypes. We concluded that changing the splicing pattern of Syk influences CRC cell viability and apoptosis, in a manner that is independent of the overall level of gene expression.

K-Ras mutations occur as an early event in about 50% of CRC cases, resulting in constitutive activation of the RAS-RAF-MEK-ERK pathway, and a subsequent resistance to anti-EGFR therapy by monoclonal antibodies Cetuximab and Panitumumab. Thus, we focused our studies on the role of Syk in HCT-116 and DLD-1 cell lines, which both bear the activating K-Ras (G13D) mutation, and which express low and high levels of Syk, respectively.

**Fig 3:**
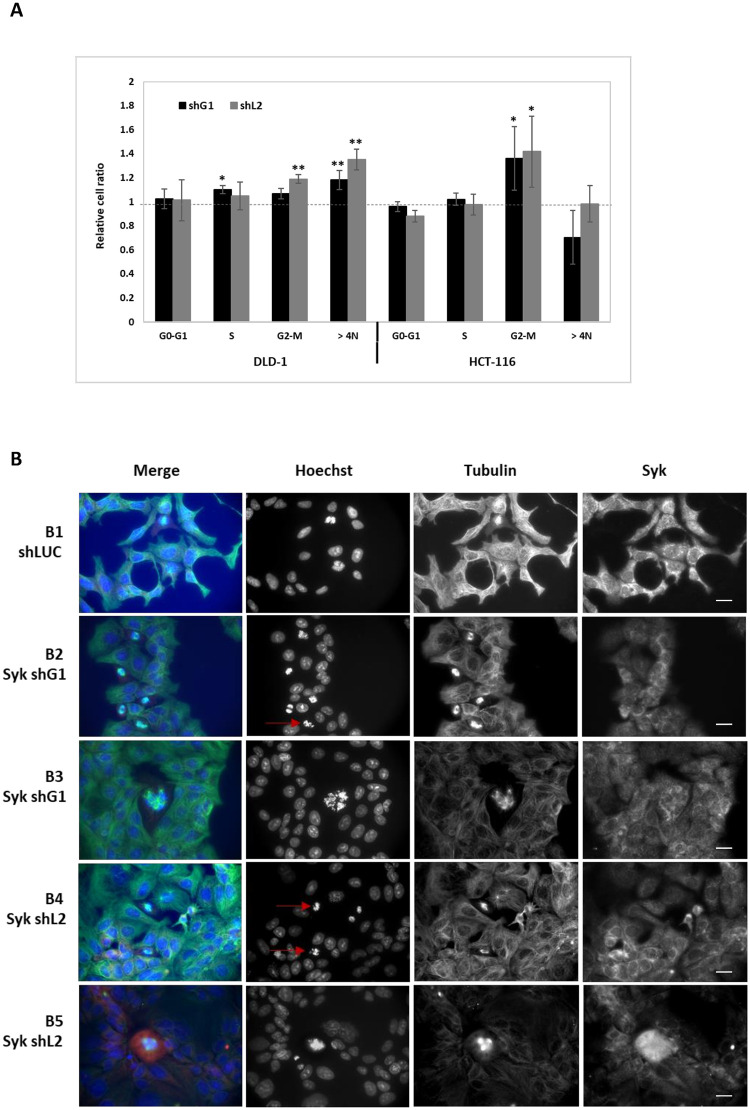
Syk (L) isoform regulates cell-cycle progression and cytokinesis. (A) DLD-1 cells were transduced with Syk shG1 and shL2 shRNAs or shLUC (control shRNA), and the cell cycle distribution of cells one week post-infection was analyzed using flow cytometry. The accumulation of transduced cells in G0-G1, S, G2-M and > 4N phases of each cell line is normalized to its control and is shown as a bar graph. The data are an average of three independent transduction experiments and error bars represent the mean ±SD (*, P<0.05; **, P<0.01). (B) Representative fluorescence microscopy images of DLD-1 cells in mitosis. DLD-1 cells were transduced with shLUC (control shRNA) (panel B1), Syk shG1 shRNA (panels B2-B3) and shL2 shRNA (panels B4-B5). One week post-infection, cells were cultured for 24 hours in Labtek glass chambers and stained with anti-Syk antibody (APC, red), anti-beta Tubulin (Alexa 488, green). Nuclei were stained with Hoechst 33342 dye (blue). Slides were analyzed visually using Zeiss Imager M2 microscope (system magnification: 60x). Two-color fluorescence merge images of representative fields show normal size nuclei and normal bipolar spindles in control cells (panel B1), while cells tranduced with Syk shG1 (panels B2) and shL2 shRNAs (panel B4) showed abnormal metaphases with aberrant accumulation of unaligned chromosomes in midzone (red arrows) and abnormal multipolar spindles, enlarged nuclei and abundant Syk expression (panels B3 and B5). Similar results were observed in three independent experiments. Scale bar: 10 μm.

Previous works reported the implication of Syk in the control of cell cycle and mitosis [9,10,28]. To further investigate the effect of Syk alternative splicing on cell proliferation, we monitored the impact of shRNA-based Syk targeting on cell-cycle progression of HCT-116 and DLD-1 cell lines. Our data showed that changing the alternative splicing of Syk led to the accumulation of cells in the G2-M phase, consistent with a defective mitosis, and to the emergence of cells with a >4N DNA content due to polyploidization through mitotic skipping (Fig 3A). This was particularly true for DLD-1 cells, which were more affected than HCT-116 in their proliferation and survival (Fig 2).

Upon the visual examination of DLD-1 cells expressing shRNA that target Syk global and isoform specific expression, we noted an increasing amount of mitotic cells with abnormal metaphases and chromosome alignment aberrations in comparison to cells expressing unrelated shRNA (Fig 3B). We also noted an increasing amount of “giant cells” displaying large and aberrant nuclei with supernumerary centrosomes and multipolar anaphase spindles confirming the role of Syk in mitotic exit and cytokinesis (Fig 3B). This mitotic arrest was particularly visible following Syk long-isoform knockdown, but was also present following Syk general knock down with global shRNA, indicating that Syk (L) controls mitotic exit and cytokinesis. Taken together, our data suggest that Syk (L) plays an important role in the cell survival and the control of the cell cycle in CRC cell lines.

### Syk inhibition increases chemotaxis and Matrigel engagement

**Fig 4:**
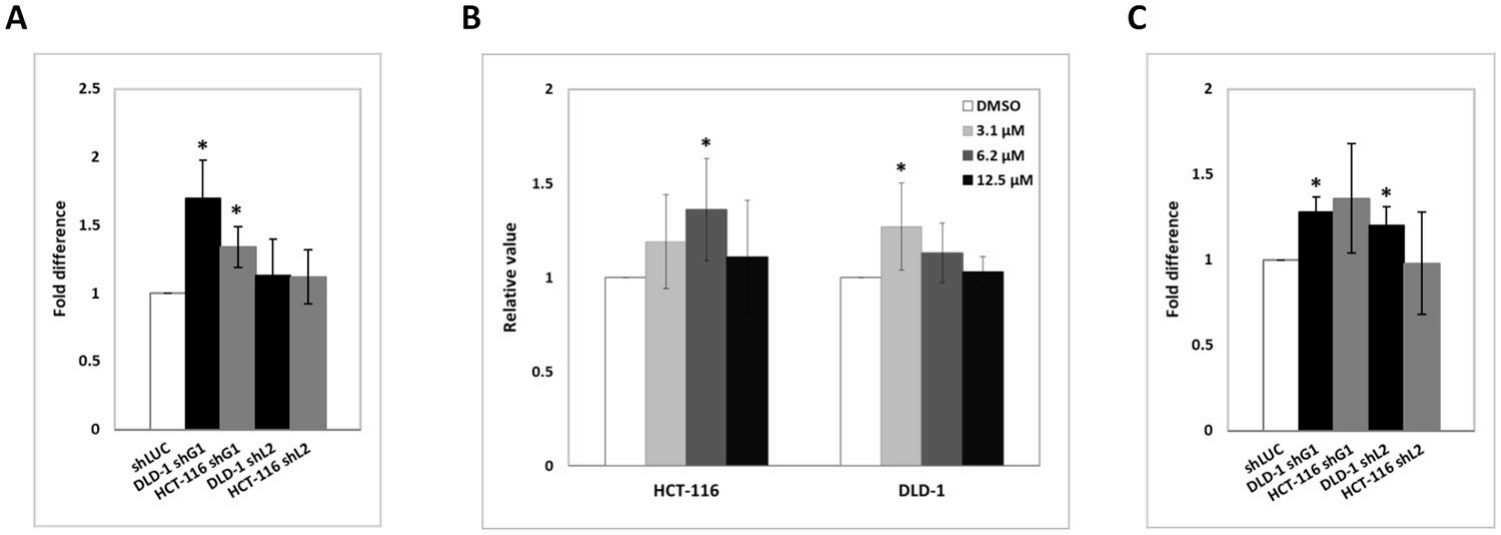
Syk inhibition increases chemotaxis and Matrigel engagement. (A) HCT-116 and DLD-1 cells (5x10^4^) transduced with shRNAs targeting global Syk (shG1) & long-isoform-specific Syk (shL2) or unrelated-transduced shRNA (shLUC), and (B) cells pretreated with Piceatannol or vehicle (DMSO) were serum starved, labelled with CellTracker dye, and migrated toward FCS in Fluoroblock chambers. (C) HCT-116 and DLD-1 cells (5x10^4^) transduced with Syk shRNAs or control shRNA were serum starved, labelled with CellTracker dye and seeded on Matrigel-coated Transwell inserts of 8 μm pore size (BD Falcon). FCS was used as chemoattractant in the Transwell lower part. After 24 hours at 37°C, migration was observed and counted microscopically. Images were obtained and total number of cells per field of view was measured using ImageJ software. Error bars represent the mean number of migrated cells in triplicate wells, representative of three independent experiments (*, P<0.05).

To investigate any correlation between Syk expression and cell motility, HCT-116 and DLD-1 cell lines were tested in a Fluoblok chemotaxis assay. We monitored the impact of Syk knockdown on cell motility, using shRNA-based Syk global and isoform specific expression. Chemomigratory ability was significantly increased in cells expressing shRNA that target Syk global expression relative to cells expressing unrelated shRNA, while Syk long-isoform knockdown did not affect cell migration ability (Fig 4A).

Next, we used the pharmacologic inhibition of Syk to further study its role in cell motility. Piceatannol, a hydroxystilbene derivative of resveratrol, preferentially inhibits the kinase activity of Syk in *in vitro* assays and is widely used as a Syk-selective inhibitor. We used a non-toxic concentration range of Piceatannol (up to 12.5 μM) commonly used in studies using human cells. As shown in Fig 4B, Piceatannol increased FCS-induced chemomigration of both HCT-116 and DLD-1 cell lines to the same manner than shRNA-based Syk global knockdown.

Cell attachment and spreading on matrix proteins is a key step in invasion. When cultured on Matrigel, many tumor cells attach and elongate, and this, together with motility, results in formation of cordlike structures and ultimately invasion. We used this experimental metastasis model to evaluate the role of Syk in colorectal carcinoma invasion and metastasis. As shown in Fig 4C, DLD-1 cell lines expressing shRNA that target Syk global expression, as well as long-isoform specific shRNA, displayed increased cell invasion compared to cells expressing control shRNA. This tendency was also observed in Matrigel invasion of HCT-116 cell lines expressing both shRNA, however, it did not reach a significant statistical value because of the high variability of this experiment.

Together, our data indicated that global Syk knock down increases cell proliferation and cell motility, while the absence of Syk (L) isoform affects cell viability and induces cell apoptosis.

### Syk expression is required for the activation of PI3K-Akt survival pathway

The epidermal growth factor receptor (EGFR) signaling pathway is commonly activated in colorectal cancer. Activation of this pathway occurs after ligand binding to EGFR, which leads to EGFR phosphorylation and oligodimerization at the plasma membrane. This in turn triggers a chain of downstream signaling events that include activation of the Ras/mitogen-activated protein kinase (MAPK) and phosphoinositide 3-kinase (PI3K)-Akt pathways that are crucial mediators of growth factor-induced proliferation and cell survival, respectively. Previous studies have shown that the presence of activating PIK3CA and Ras mutations in CRC cell lines result in constitutive activation of these pathways downstream of EGFR signaling.

Considering the presence of activating K-Ras (G13D) mutation in both HCT-116 and DLD-1 cell lines, as well as PIK3CA (H1047R) mutation in HCT-116 cell line, we investigated the effects of Syk targeting on EGFR ligand-induced activation of Erk and Akt. For this purpose, HCT-116 and DLD-1 cells transduced with shRNAs that target Syk global and long-isoform specific expression or firefly luciferase (shLUC; control) were serum starved, then treated with EGFR ligand, EGF. Cell lysates were subsequently analyzed by immunoblotting using activation state-specific antibodies for Erk and Akt (S1A Fig). Our data showed that HCT-116 cells display constitutively active Erk1/2 and Akt proteins, shown by their phosphorylation state, which remained comparable between untreated and EGF-treated cells. However, EGF treatment induced the phosphorylation of Erk1/2 and Akt in DLD-1 cell line (p-Erk; p-Akt; shLUC: -/+ EGF).

In both cells lines, shRNA-mediated Syk depletion did not affect the phosphorylation levels of Erk1/2 which remained comparable between EGF-treated and untreated cells (p-Erk; shG1, shL2: -/+ EGF). However, targeting Syk global and Syk (L) isoform affected the level of constitutive phosphorylation of Akt in HCT-116 cells, as well as the level of EGF-induced phosphorylation of Akt in both cell lines (p-Akt; shG1, shL2: -/+ EGF) (S1A Fig).

Cetuximab is a chimeric IgG1 monoclonal antibody that targets the extracellular domain of EGFR, blocking ligand binding to the receptor and leading to the inhibition of EGFR signaling. The pretreatment of the cells with Cetuximab attenuated the mitogenic effect of EGF and inhibited the phosphorylation of Akt and Erk1/2 in both cell lines; however, the phosphorylation of Erk1/2 in HCT-116 cell lines was mildly affected by Cetuximab. In all cases, the expression level of EGFR in the different cell lines, analyzed by western blot (S1A Fig) and by FACS (data not shown) was not affected. Together, these results showed that Syk (L) expression is required for the sustained and/or growth factor-induced activation of the PI3K/Akt pathway.

### EGF exerts opposite effects on Syk alternative splicing

Growth factors can affect cell survival by regulating the activity of splicing modulators and the splicing of kinases such Syk. We investigated the effect of short-term EGF treatment on the alternative splicing of Syk in HCT-116 and DLD-1 cell lines *in vitro*. Our data showed that EGF exposure increases exon 9 inclusion and the expression levels of Syk (L) isoform in DLD-1 cell line, while it exerts the opposite effect in HCT-116 cell line by inducing exon skipping and the increase in Syk (S) isoform expression (S1B Fig). Therefore, we concluded that mitogenic signaling regulates both the amount and the ratio of Syk splicing isoforms but in a manner dependent on cell background.

Considering the accumulating evidence supporting the role of Syk (L) isoform in CRC cell survival and cell cycle regulation, we further investigated the role of Syk downstream of EGFR signaling. For this, we performed Syk “pull-down assays” on lysates of serum-starved and EGF-stimulated DLD-1 cells, and we analyzed the components of the captured complexes by high-resolution mass spectrometry to identify Syk interactomes. Our data showed that upon EGF treatment, there is an increased association of Syk with ligands that are implicated in the control of apoptosis, and in the regulation of mitosis and cell cycle G2/M checkpoint, such as Nuclear death domain protein p84N5, which activates a G2/M cell cycle checkpoint prior to the onset of apoptosis [29]; RAD50, which regulates mitotic progression independent of DNA repair functions [30]; and cell division cycle protein 23 homolog (CDC23), a protein component of anaphase-promoting complex (APC) [31] (S1C Fig). These data confirm the implication of Syk in the control of the cell cycle and the apoptosis of CRC cell lines, which is enhanced under the influence of oncogenic growth factors such as EGF.

### Syk Long isoform expression is associated with tumorigenesis in human CRC tissues

**Fig 5:**
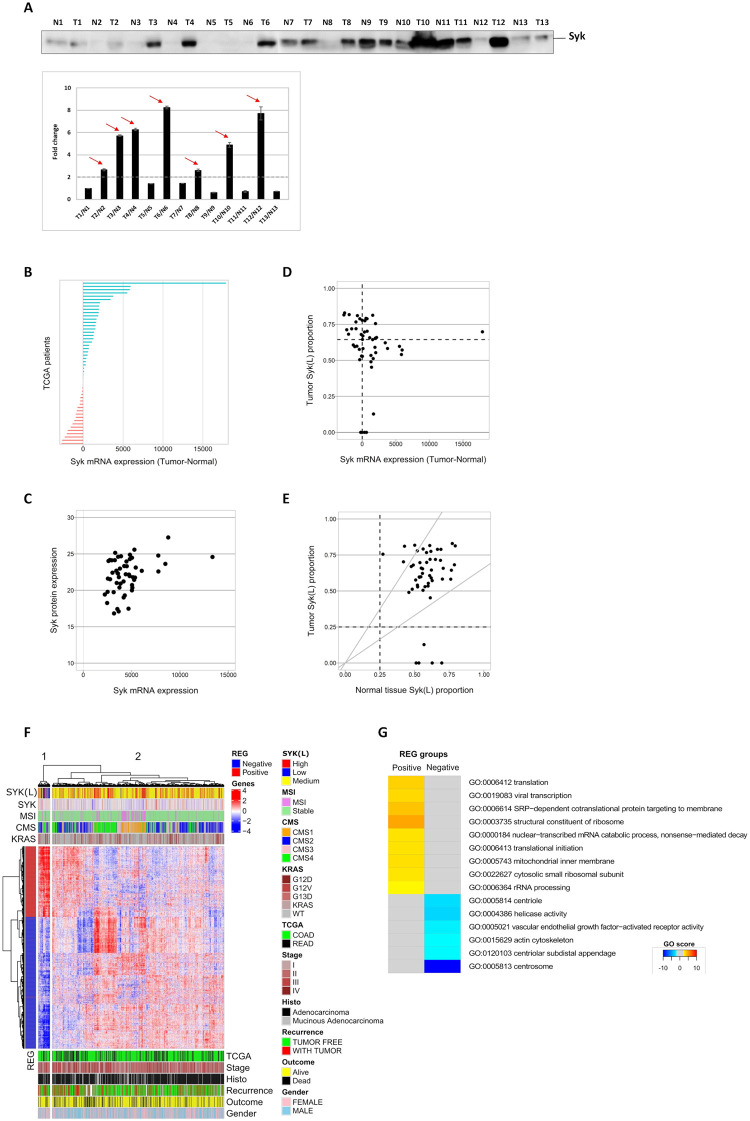
Syk (L) isoform expression is associated with tumorigenesis in human CRC tissues. (A) *Top panel*- Western blot analysis of the protein extracts of primary colorectal tumors of 13 untreated patients. T: Tumor tissue and N: normal adjacent tissue of the same patient. *Bottom panel*- Densitometric quantification of immunoblot analyses of Syk expression in tumor tissues over the corresponding normal tissues. Red arrows indicate the tumor samples in which the ratio of Syk expression is at least two fold higher compared to the normal tissues. (B) Analysis of Syk mRNA differential gene expression in tumor and normal tissue of N = 50 patients from TCGA database COAD and READ cohorts. (C) Correlation between Syk mRNA and Syk protein expression in tumor tissues of N = 52 patients. (D) Analysis of Syk (L) isoform expression over the total Syk transcripts in tumor tissues of N = 50 patients. The horizontal dashed line indicates the median of PSI value in tumor samples. (E) High proportion of Syk (S) isoform are only present in tumors. The PSI values were compared in 50 patients presenting both tumor and normal tissue value. The above gray curve corresponds to the 1.5 limit of Syk (L) expression in tumor sample over normal tissue ratio, whereas the bottom gray curve is the reverse one. Dashed line figures out the 0.25 PSI limit defining low PSI samples. (F) Differentially expressed genes between low and high PSI tumors. Heatmap represents the z-score transformed gene expression values. Clinical data on TCGA patient were recovered from [26]. CMS groups were recovered from [32]. K-Ras mutations were downloaded from cbioportal. KRAS means all mutations except G13D, G12V and G12D, which are the most prevalent ones in TCGA patients. The relationship between PSI expression and MSI score is according to [27]. The REG value is calculated as the mean of z-score of gene expression in the low-PSI cluster of 40 samples. (G) GO terms enrichment between positive REG (583 genes) and negative REG genes (1070 genes) as defined in panel F. Red colors correspond the upregulated genes and blue colors to down regulated genes in the PSI low associated cluster of 40 patients. The score is the log10 of the corrected p-value of enriched terms in each group. The sign of the score is the sign of the REG value (i.e. mean of z-score in the low-PSI group).

In a previous study, we reported a gene expression signature associated with treatment response in advanced CRC patients [24]. Thirteen pairs of colon primary tumors (CT) and normal colon mucosas (CN) were collected at the time of surgery, before chemotherapy. We used these samples to determine the profile of Syk expression in primary human colorectal tumors. For this purpose, we performed western blot analysis of protein extracts from tumor tissues of these 13 untreated patients and we compared the expression of Syk protein to that of normal adjacent tissues of the same patient. Our data showed that Syk protein levels were higher in 50% of the analyzed human tumors compared to the normal tissues (Fig 5A). However, we were not able to analyze the ratio of Syk (L) versus Syk (S) isoforms due to the absence of long isoform-specific antibodies.

Next, we conducted an analysis integrating Syk global and isoform-specific expression data, as well as clinical meta-data of 622 CRC patients (COAD and READ cohorts) from The Cancer Genome Atlas (TCGA). This analysis showed a high Syk mRNA expression score in CRC tumors (S2A Fig). Accordingly, the comparison of mRNA expression levels of global Syk in tumor and normal tissue of same patients (N = 50) revealed an upregulation of Syk gene expression in two thirds of the primary tumors (Fig 5B). Furthermore, high mRNA expression was associated with high protein level in TCGA tumors but the opposite was not always true (N = 52) (Fig 5C). This is possibly due to Syk protein turnover differences.

We next analyzed Syk splicing variants expression in CRC tumors using the COAD and READ cohorts of TCGA SpliceSeq database. This database reports the PSI (percent-splice-in) values for the splice events in tumor and normal adjacent tissues [25] (S2B Fig). We extracted PSI values for exon 9 of Syk gene, which correspond to the ratio of Syk (L) isoform expression over the total Syk transcripts (S2C Fig). Since Syk (L) isoform results from the inclusion of exon 9 in Syk gene, the PSI value reflects the proportion of Syk (L) isoform expression in patient’s tissue samples.

The comparison of PSI values of normal and tumor tissues of the same patients (N = 50) revealed that both normal and tumor tissues present high PSI values reflecting the dominant expression of Syk (L) isoform (S2B Fig). Our analysis showed that most tumor samples express higher amounts of Syk (L) than Syk (S) mRNA (PSI values > 0.5, N = 43/50) (Fig 5D). Moreover, the PSI values of tumor tissues in which Syk expression is down-regulated remain high, suggesting a dependence of tumors to Syk (L) isoform expression. Interestingly, 10% of the analyzed tumor tissues (N = 5/50), and none of the normal tissues, have low PSI values (PSI < 0.25), reflecting the expression of a high proportion of Syk (S) isoform (Fig 5E). The level of expression of global Syk in these tumor samples is comparable to that of the adjacent normal tissue (Fig 5D).

To further characterize this specific subpopulation and the consequences of Syk PSI value on tumor biology, we classified 619 CRC tumor samples based on gene expression data. We first classified samples as low PSI (PSI<0.25; 20 samples), medium PSI (452 samples) and high PSI (PSI>0.75; 147 samples) tumors. Next, we selected 1653 genes differentially expressed between the low and high PSI samples and we analyzed tumor subpopulations by hierarchical clustering (Fig 5F). Interestingly, we identified a small cluster of 40 samples designated as low PSI-like tumors. This cluster includes 17 low PSI, 13 medium PSI and 10 high PSI tumors. This group of 40 samples forms a specific cluster associated with a large set of 583 strongly over-expressed genes and 1070 down-regulated genes, compared to other tumor samples (REG value). The REG positive and negative groups overlap the two clusters obtained from unsupervised analysis. Our analysis showed that 495 genes from the first main cluster associated with REG positive values were related to translation and mitochondria, whereas 1158 genes from the second main cluster related to REG negative values were associated with centriole and centriolar functions (Fig 5G). Finally, this group of PSI-low tumors was not related to any classical CRC classification such as MSI status, CMS molecular classification, K-Ras mutational status, or TNM stage, nor to Syk total expression level.

Taken together, our analysis suggests the dependence of CRC tumors to the pro-survival Syk (L) isoform, except in the new identified subgroup of low PSI tumors in which gene reprograming has taken place to overcome the effect of Syk (L) down-regulation.

### Treatment of CRC cell lines with C-13, an original non-enzymatic inhibitor of Syk

In our previous works, we developed an original approach for the identification of protein-protein interaction inhibitors of Syk [33]. This led to the discovery of new classes of non-enzymatic inhibitors of Syk with improved selectivity profile and which bind to a cavity located at the interface between the SH2 domains and the linker domain of Syk (Patent No WO2009133294). Among the newly isolated scaffolds, compound C-13 showed a high potential for the inhibition of mast cell degranulation *in vitro* (Syk IC50 = 2 μM) and for the prevention of anaphylactic shock in mice using oral delivery route *in vivo* (Syk IC50 = 110 mg/kg) [23].

**Fig 6:**
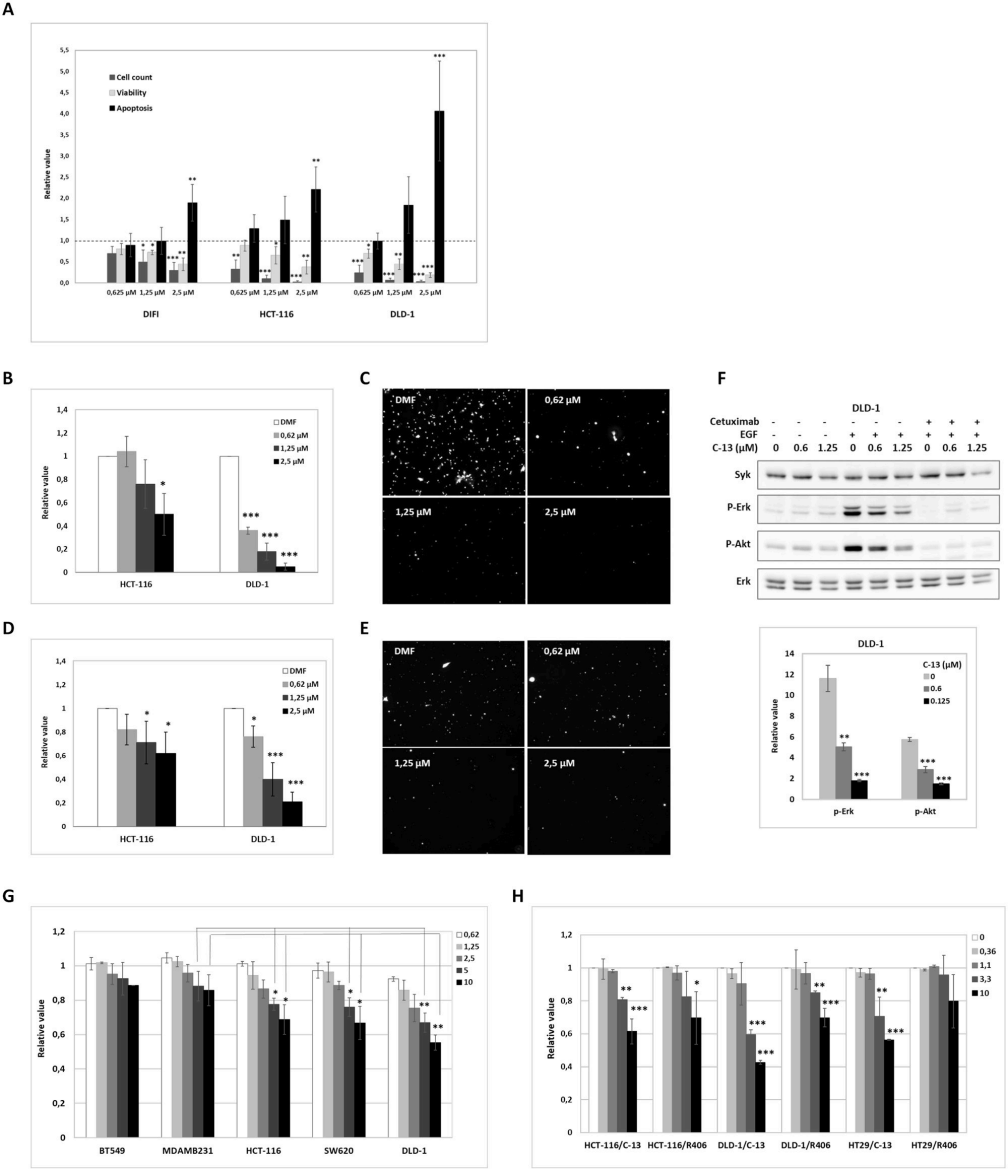
Treatment of CRC cell lines with C-13, an original non-enzymatic inhibitor of Syk. (A) Cells were exposed for 2 hours to various concentrations of C-13 and the impact of C-13 on cellular phenotype evaluated 72 hours post-treatment by: cell proliferation, viability and apoptosis, and normalized against DMF (vehicle)-treated HCT-116, DLD-1 and DIFI CRC cells. Data are the mean ±SD (error bars) of at least three independent experiments (*, P<0.05; **, P<0.01; ***, P<0.001; compared with control). (B-C) Cells pretreated for 2 hours with C-13 or vehicle (DMF) were serum starved, labelled with CellTracker dye, and migrated toward FCS in Fluoroblock chambers. (D-E) Cells pretreated with C-13 or vehicle (DMF) were serum starved, labelled with CellTracker dye and seeded on Matrigel-coated Transwell inserts of 8 μm pore size (BD Falcon). FCS was used as chemoattractant in the Transwell lower part. After 24 hours at 37°C, migration was observed and counted microscopically. Images were obtained and total number of cells per field of view was measured using ImageJ software. Representative microscopy images are shown in Fig 6C and 6E. Error bars represent the mean number of migrated cells in triplicate wells, representative of three independent experiments (*, P<0.05; **, P<0.01; ***, P<0.001; compared with control). (F) DLD-1 cells pre-treated with the indicated concentrations of compound C-13 or DMF (vehicle) for 2 hours, were incubated with the recombinant human EGF (50 ng/mL) or a combination of EGF (50 ng/mL) + Cetuximab (50 μg/mL) for 10 minutes. *Top panel*- Protein extracts from cell lysates were analyzed by western blot using the indicated antibodies. *Bottom panel*- Densitometric quantification of immunoblot analyses of phospho-Erk (+EGF) versus phospho-Erk (-EGF) over control and phospho-Akt (+EGF) versus phospho-Akt (-EGF) over control. G) Syk-positive CRC cell lines DLD-1, SW620 and HCT-116; and Syk-negative breast cancer cells lines MDAMB231 and BT549 were exposed to various concentrations of C-13 for 72 h; (H) HCT-116, DLD-1 and HT29 cell lines were treated in parallel with C-13 (vehicle DMF) and R406 (vehicle DMSO) for 72 h; and cell viability was measured using CellTiter-Glo Luminescent cell viability assay (Promega). The relative changes were calculated and used to generate the bar graphs. The error bars represent the variation observed in three biological and three technical replicate. (*, P<0.05; **, P<0.01; ***, P<0.001; compared with Syk-negative MDAMB231 cell line for panel G; compared with vehicle for panel H).

We studied the impact of compound C-13 on the biology of CRC cell lines. We exposed a wild type (DIFI), and two K-Ras mutant (HCT-116, DLD-1) CRC cell lines to various concentrations of C-13 for 2 hours and then measured cell proliferation, viability and apoptosis 72 hours post-treatment. Our data showed that C-13 inhibits cell proliferation and induces apoptosis of the cells (Fig 6A) in a manner that mimic shRNA Syk (L) isoform-specific targeting but not total Syk targeting by shRNA (Fig 2). Moreover, C-13 inhibits FCS-induced chemomigration (Fig 6B and 6C) and invasion (Fig 6D and 6E) of CRC cell lines in a concentration-dependent manner. Interestingly, the cytotoxic effect of C-13 on DLD-1 cell line was higher than on the two other cell lines, and was confirmed by western blot analysis which showed that EGF-induced phosphorylation of Erk1/2 and Akt were both markedly reduced in DLD-1 cells pre-treated with C-13 (Fig 6F). FACS analysis showed that the level of membrane expression of EGFR in the cell lines was not affected by C-13 (data not shown). To demonstrate that the effect of C-13 on cells viability is indeed due to a Syk-specific inhibition and not to an off-target interaction, we used Syk-positive CRC cell lines DLD-1, SW620 and HCT-116, and Syk-negative breast cancer cells lines MDAMB231 and BT549 as control. Cells were exposed to various concentrations of C-13 for 72 h and cell viability was measured. Our data showed that C-13 specifically exerts cytotoxic effects on Syk-positive cell lines, while Syk-negative cell lines viability was unaffected (Fig 6G).

Finally, we compared C-13 with R406 (the active metabolite of Fostamatinib R788), a specific, ATP-competitive inhibitor of Syk (Syk IC50 = 41 nM) that has shown efficacy in the treatment of autoimmune diseases [34] and in preclinical leukaemia studies [35,36]. We compared the effects of increasing concentration of the two drugs on the survival of HCT-116, DLD-1 and HT29 CRC cell lines that express low to high levels of Syk, respectively (Fig 6H). Our data showed that despite a 300 fold lower *in vitro* affinity for Syk compared to R406 (4 μM for C-13 [23] versus 12 nM for R406 [37]), C-13 affects the viability of Syk-positive CRC cell lines in a more efficient manner than R406. Together with the effect of Piceatannol on cell migration (Fig 4B), our data demonstrate that compounds that target Syk catalytic activity are less efficient than compound C-13 on the inhibition of CRC cell lines proliferation and motility.

### Compound C-13 attenuates tumor growth in CRC xenograft mice model

We established two *in vivo* mice models to examine the anti-tumor effect of C-13 on CRC tumor growth. For these experiments, xenografts in athymic nude mice were established by subcutaneous injection of 1x10^6^ DLD-1 cells and the drug administration was initiated when tumors reached the volume of 40–70 mm3. For the intraperitoneal (IP) and oral administrations, we referred to our previous *in vivo* studies with C-13 in order to choose nontoxic doses of this compound and to minimize adverse effects [23].

**Fig 7:**
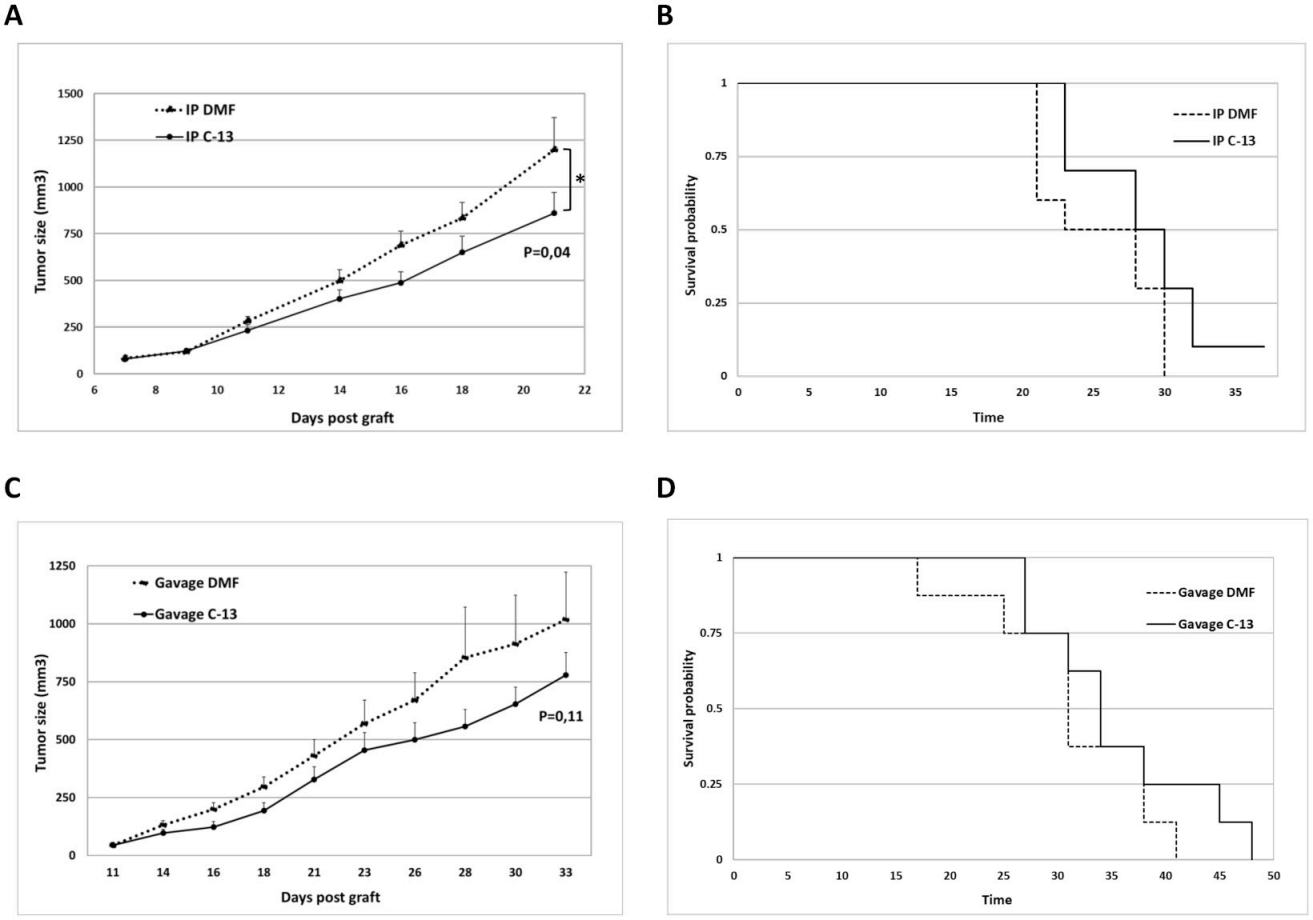
C-13 inhibits tumor growth of xenografts in Athymic Nude mice. (A) Effect of intraperitoneal treatment with C-13 (0.35 mg/kg) or DMF (vehicle) (3 times/week) on tumor growth in Athymic Nude mice xenografted with 1x10^6^ DLD-1 CRC cells (n = 10 mice/group). (B) Kaplan-Meier survival curves of the mice described in B. C) Effect of oral administration of C-13 (100 mg/kg) or DMF (vehicle) (3 times/week) on tumor growth of Athymic Nude mice xenografted with 1x10^6^ DLD-1 CRC cells (n = 7 mice/group). D) Kaplan-Meier survival curves of the mice described in C.

We first investigated the effect of IP administration of C-13 on tumor growth. For this purpose, two groups of 9 mice received an IP administration of compound C-13 at a dosage of 0.35 mg/kg or DMF alone (vehicle). The drug was administered three times a week over 3 weeks. The tumor sizes were measured and recorded every 2 days, and tumor growth curves analyzed for each group. At the given dose, there was a significant decrease in tumor growth in the group treated with C-13 compared to the control group treated with vehicle only (p = 0.04) suggesting C-13’s potently inhibitory effect against tumor growth (Fig 7A). Kaplan-Meier survival analysis confirmed this observation (Fig 7B). We did not evidence any apparent toxicity of the treatment and body weight growth and physical activity of mice were similar in both groups. In a second *in vivo* experiment, we investigated the effect of the oral administration of C-13 on the tumor growth of DLD-1 cells xenografts in athymic nude mice. For this purpose, 100 mg/kg of C-13 or DMF alone (vehicle) were administered by gavage to two groups of 7 mice xenografted with DLD-1 cells, three times per week for 3 weeks. As shown in Fig 7C and 7D, there is a decrease in tumor growth of the group of mice treated with C-13 compared to the control group, although we did not reach a significant statistical value (p = 0.11).

Together, our *in vivo* data confirm that C-13 is a promising and original Syk-specific molecule, with potential applications in the treatment of K-Ras-mutated CRC.

## Discussion

Syk mRNA or Syk protein expression have been both positively and negatively associated with tumorigenesis. However, in most studies, only global expression of Syk was measured leaving questions regarding the prognostic value of the splice variants in different cancer types. Accumulating studies have attributed the differences in Syk behavior to the differential expression of Syk (L) and Syk (S): while the long isoform is predominantly nuclear, the short isoform is only expressed in the cytoplasm.

We have opted for shRNA approach as a tool to evaluate the effects of general knockdown of Syk as well as changing the splicing pattern of Syk, on the biology of HCT-116 and DLD-1 cell lines, which both bear the activating K-Ras (G13D) mutation. We first performed functional studies to evaluate the impact of Syk expression silencing on the viability of CRC cells, as well as their motility. Our findings presented herein showed that global knockdown of Syk increased cell proliferation and cell motility, while targeting Syk (L) isoform altered cell survival and induced the apoptosis of CRC cell lines (Figs 2 and 4). Our results correlate with the findings of Pinos et al. [6] who reported that ovarian and colorectal cancer cell lines are most sensitive to the modulation of Syk alternative splicing rather than the global knockdown of Syk expression which does not mimic the effects of alternative splicing on cell survival and apoptosis.

Other groups have previously reported the important role of Syk in cell cycle regulation through Syk’s centrosomal kinase activity [9], and as an activator of the G2 checkpoint [10]. Our data show that Syk (L) deficiency is associated with mitotic defects, leading to the accumulation of cells in the G2-M phase of cell cycle, and to the emergence of cells with a >4N DNA (Fig 3A). This is illustrated by the presence of cells with multipolar anaphase spindles, chromosome missegregation and polyploidization through mitotic skipping (Fig 3B). Accordingly, high-resolution mass spectrometry analysis of Syk interactome in DLD-1 cell line showed the association of Syk with ligands implicated in the control of apoptosis, and in the regulation of cell cycle (S1C Fig).

Taken together, our data suggest that Syk (L) isoform plays a key role in the control of cell cycle and in the survival of CRC cell lines.

Immunofluorescence analysis of CRC cells transduced with shRNA that target Syk revealed a higher cytoplasmic labelling of Syk in the cells undergoing mitosis, particularly in cells that underwent Syk long-isoform knockdown, compared to shLUC-transduced cells (Fig 3B). These observations suggest that targeting Syk (L) isoform may lead to a temporary increase of Syk (S) isoform expression, and to its accumulation. Indeed, qPCR and RT-PCR analysis of Syk isoforms expression levels showed that shRNA-mediated targeting of Syk (L) isoform leads to increased expression of Syk (S) isoform (Fig 1C and 1D). The abnormal accumulation of Syk (S) isoform in these cells may also be due to a defect in its ubiquitination and proteasomal degradation [38].

Several studies have reported the pro-survival role of Syk through Syk-dependent activation of PI3K/Akt pathway (reviewed in REF [3]). The expression of Syk is a signature of K-Ras-dependent lung and pancreatic cancer cell lines, where the knockdown of Syk expression or inhibition of Syk activity results in the apoptosis of the cells [39]. K-Ras mutations occur at high frequencies in colon cancer cells, and they result in constitutive activation of signaling pathways downstream of EGFR, including Ras/MAPK and PI3K-Akt pathways. Our data showed that the EGF-induced phosphorylation of Akt was inhibited in HCT-116 and DLD-1 cell lines expressing shRNA that target Syk global and Syk (L) isoform expression, confirming the implication of Syk in the sustained activation of pro-survival PI3K/Akt pathway.

In order to explore the prognostic role of Syk variants in colorectal cancer patients, we analyzed the TCGA database related to the expression of Syk in tumors of 622 patients of COAD and READ cohorts. Our analysis showed a high Syk mRNA expression score in CRC tumors (S2A Fig) suggesting a tumor promotor role of Syk in CRC. This observation was confirmed at the protein level by western blot analysis of global Syk expression in pairs of colon primary tumors and normal colon mucosas in a cohort of 13 patients, which showed overexpression of Syk in 50% of the colorectal tumors (Fig 5A). Lee et al. have previously analyzed the TCGA database by integrating multiple genomic features as well as the clinical meta-data available in 2013 [40]. Among the candidate genes that robustly delineated advanced clinical stage, Syk had the second highest score by mRNA expression and methylation features. The expression of Syk was also associated with the N status (N = regional lymph node cancer involvement) suggesting a possible role of Syk in colorectal cancer metastatic progression.

Our analysis of the TCGA SpliceSeq database provided additional data related to the expression of Syk splicing variants in CRC tumors. The analysis of the PSI value calculated as the ratio of Syk (L) isoform over the total Syk transcripts, showed that Syk (L) is highly expressed in the majority of the analyzed tumors (Fig 5D). Interestingly, Syk (L) remains expressed in tumors in which global Syk expression is downregulated, suggesting the dependence of tumors to Syk (L) isoform (Fig 5D). Our analysis also showed that only tumor tissues express a high proportion of Syk (S) isoform (N = 20) (Fig 5E).

Next, we studied the differential gene expression of 1653 genes between low PSI tumors (20 samples) and high PSI tumors (147 samples). This led to the identification of a small cluster of 40 samples that contains 17 of the 20 low PSI tumors, together with 13 medium PSI and 10 high PSI tumors (Fig 5F). This group of 40 samples designated as “low PSI-like tumors”, is forming a specific cluster associated with overexpressed genes related to translation and mitochondria, and down regulated genes implicated in the centrosome organization (Fig 5G). These features are commonly shared with CRC cell lines in which shRNA-mediated targeting of Syk (L) leads to increased Syk (S) expression (Fig 1C and 1D) and to defective mitosis (Fig 3), suggesting the existence of a CRC cells background in agreement with low expression of Syk(L) isoform.

The supervised analysis of the clinical data available for 619 over 622 tumors did not reveal any significant relationship between Syk variants expression and either K-Ras mutations, CMS tumor types or MSI level. However, 8/20 low PSI tumors are classified among CMS4 tumor type, and that low PSI tumors present high frequency of K-Ras mutations (4/14; fisher test p-value 0.0474). This tendency was also observed in the small cluster of 40 “low PSI-like tumor” samples (18/33) compared to the large cluster of remaining tumor tissues (198/414) for which the information on K-Ras mutations were available.

Previous studies showed that EGF regulates the alternative splicing through the activation the serine-arginine-rich (SR) family and RNA-binding proteins, leading to the induction of exon inclusion pathways [41–43]. EGF also regulate the expression and the activity of negative regulatory proteins such as heterogeneous nuclear ribonucleoprotein (hnRNP) families [44], among which hnRNP-K protein was shown to regulate the splicing pattern of Syk [45].

We studied the effect of short-term EGF treatment on Syk alternative splicing. We found Syk exon inclusion and increased Syk (L) expression in DLD-1 cells, while EGF treatment led to Syk exon exclusion and the increase in Syk (S) isoform expression in HCT-116 cells (S1B Fig), suggesting that tumors that are responsive to growth factors may express high levels of Syk (L) isoform. However, tumors that express hyperactivating mutations and that are less responsive to extracellular signals, express higher levels of Syk (S) isoform due to exon exclusion.

Together, these observations underline the need to understand the links between Syk alternative splicing and tumor behavior in response to growth factors and stress signals, influenced by the mutational status of tumors.

Syk plays a major role to support cell survival in multiple B cell-derived lymphomas where it is an important mediator of BCR-dependent signaling. Syk is also essential for FcεRI-triggered mast cell activation, and in type II and type III hypersensitivity reactions mediated by Fcγ receptors. Fostamatinib (R788), a soluble pro-drug form of the Syk inhibitor R406, has been used as anti-inflammatory therapeutics in phase II clinical trials for the treatment of rheumatoid arthritis (RA) [34], in B-cell lymphocytic leukemia (B-CLL) [35] and in diffuse large B cell lymphoma (DLBCL) patients [46] with a positive response to therapy. A pro-survival role for Syk is also seen in retinoblastoma where the addition of the Syk inhibitor BAY61-3606 to the chemotherapeutic regimen used to treat retinoblastoma in a mouse model significantly improves outcomes [17].

In our previous works, we have isolated compound C-13, a small molecule that is a non-enzymatic inhibitor of Syk [23]. C-13 inhibited early and late mast cell responses induced by FcεRI aggregation *in vitro* (IC50 = 2 μM). *In vivo*, the oral administration of C-13 inhibited anaphylactic shock in Balb-C mice (IC50 = 110 mg/kg).

We studied the effects of compound C-13 on the biology of CRC cell lines. Our data showed that compound C-13 exerts cytotoxic effects on CRC cells by inhibiting their proliferation and their motility (Fig 6). Moreover, C-13 affects the survival of CRC cells by inducing their apoptosis, to the same manner than the shRNA-mediated silencing of the long isoform of Syk (Fig 2). Accordingly, EGF-induced signal transduction by the PI3K/Akt and Ras/Erk pathways were inhibited by C-13 in a concentration dependent manner, as shown by the markedly decreased phosphorylation of Akt and Erk 1/2 in DLD-1 cells treated with C-13 (Fig 6F).

The specificity of C-13 towards Syk was further confirmed by its cytotoxic effects on Syk-positive CRC cell line, while Syk-negative breast cancer cell line’s survival were not significantly affected by C-13 (Fig 6G). Moreover, despite a 300 fold lower *in vitro* affinity for Syk in comparison to the Syk enzymatic inhibitor R406, C-13 had a stronger inhibitory effect on CRC cell lines viability (Fig 6H). *In vivo*, the intraperitoneal and oral administration of C-13 inhibited the tumor growth of Athymic Nude mice DLD-1 xenografts (Fig 7), without any apparent toxic effects over a period of 3 weeks, suggesting C-13’s potently inhibitory effect against tumor growth.

Thus, compound C-13 and its derivatives may have promising applications in the treatment of CRC and other solid and hematological cancer diseases (Patent application No EP 22306229). Indeed, C-13 as a “hit” molecule from a high throughput screen may undergo medicinal chemistry optimization to identify “lead” drug-like compounds with higher solubility, affinity and selectivity, leading to new classes of orally available non-enzymatic inhibitors of Syk.

C-13 binds to a cavity located at the interface of the two SH2 domains and the intradomain A of Syk, a rather unique interaction area that is specific to Syk, suggesting that this molecule acts as a selective inhibitor of Syk [23]. C-13 may inhibit the interaction of Syk with some of its macromolecular substrates, either directly because part of C-13 occupies a surface where a Syk partner could make direct contact and/or through an allosteric effect. The molecular mechanism of action of compound C-13 on CRC cells function is currently under investigation.

In summary, our data presented herein suggest that Syk (L) isoform may be a target for the treatment of the majority of colorectal cancers, except a small group of tumors that express high levels of Syk (S) isoform and a strong decrease of Syk (L) isoform expression. Considering the key role of Syk (L) isoform, the identification, for the first time, of lower Syk (L) expression tumors was intriguing. Further studies will be needed to characterize genomic and metabolic modifications associated to the Syk(L) low phenotype and related CRC tumors.

## Supporting information

S1 Checklist The ARRIVE guidelines 2.0: Author checklist. (PDF)

S1 Fig The implication of Syk isoforms in CRC survival and cell cycle regulation upon EGF stimulation. (A) The implication of Syk isoforms in CRC survival. Cells transduced with Syk shG1, Syk shL2 and shLUC (control) shRNAs were serum-starved, then treated with the recombinant human EGF (50 ng/mL) or a combination of EGF (50 ng/mL) + Cetuximab (50 μg/mL) for 10 minutes. *Top panel*- Protein extracts from cell lysates were analyzed by western blot using the indicated antibodies. *Bottom panel*- Densitometric quantification of immunoblot analyses of Syk, phospho-Akt, phospho-Erk and EGFR expression over control. (B) EGF promotes Syk alternative splicing by exon 9 inclusion / exclusion. HCT-116 and DLD-1 cell lines were serum starved for 16 h, then treated with EGF (50 ng/mL) for 15 minutes. The qPCR relative expression values for the overall gene expression Syk (L+S), long splice isoform Syk (L) and short splice isoform Syk (S) were normalized against the qPCR values obtained for untreated cells used as control (NT). The bar graph shows the changes in Syk splicing upon short-term EGF treatment. Error bars represent the mean ± SD of three independent experiments (*, P<0.05; **, P<0.01). (C) Identification of Syk interactome in serum starved and EGF-treated DLD-1 cells, by Mass spectrometry. Protein lysates from serum-starved and EGF-treated DLD-1 cells were incubated with agarose-conjugated anti-Syk 4D10 monoclonal antibody and agarose-conjugated mouse IgG (control) for 2 hours at 4°C. Beads were washed in lysis buffer, then they were resuspended in 1x Laemmli sample buffer and the protein contents were analyzed by SDS-PAGE. The table represents protein contents of Syk pulldowns in the absence of EGF (-EGF) and in the presence of EGF (+EGF) that scored over 15 and that were absent in the control IgG pulldowns. For data analysis, peptide and protein identifications were performed in Uniprot/Swiss- Prot2016_01 database by ProteinPilotTMSoftware V 4.5 (Sciex).
(TIF)

S2 Fig Syk splicing variants expression in CRC tumors. (A) Syk mRNA expression score in CRC tumors of The Cancer Genome Atlas (TCGA) (https://www.proteinatlas.org/ENSG00000165025-SYK/summary/rna). (B) Syk splicing variants expression in COAD and READ cohorts of the TCGA SpliceSeq database. The PSI (percent-splice-in) values correspond to the ratio of Syk (L) isoform expression over the total Syk transcripts calculated for the tumor tissues (red) and normal adjacent tissues (green) of the same patient [25]. (C) Genomic map of human Syk locus on chromosome 9 q22-2.
(TIF)

S1 Raw images (PDF)
