# Supplementary figures and images for: Targeting the splicing isoforms of spleen tyrosine kinase affects the viability of colorectal cancer cells
Source: PLoS One. 2022 Sep 14;17(9):e0274390. doi: 10.1371/journal.pone.0274390 (PMC9473616; doi:10.1371/journal.pone.0274390)

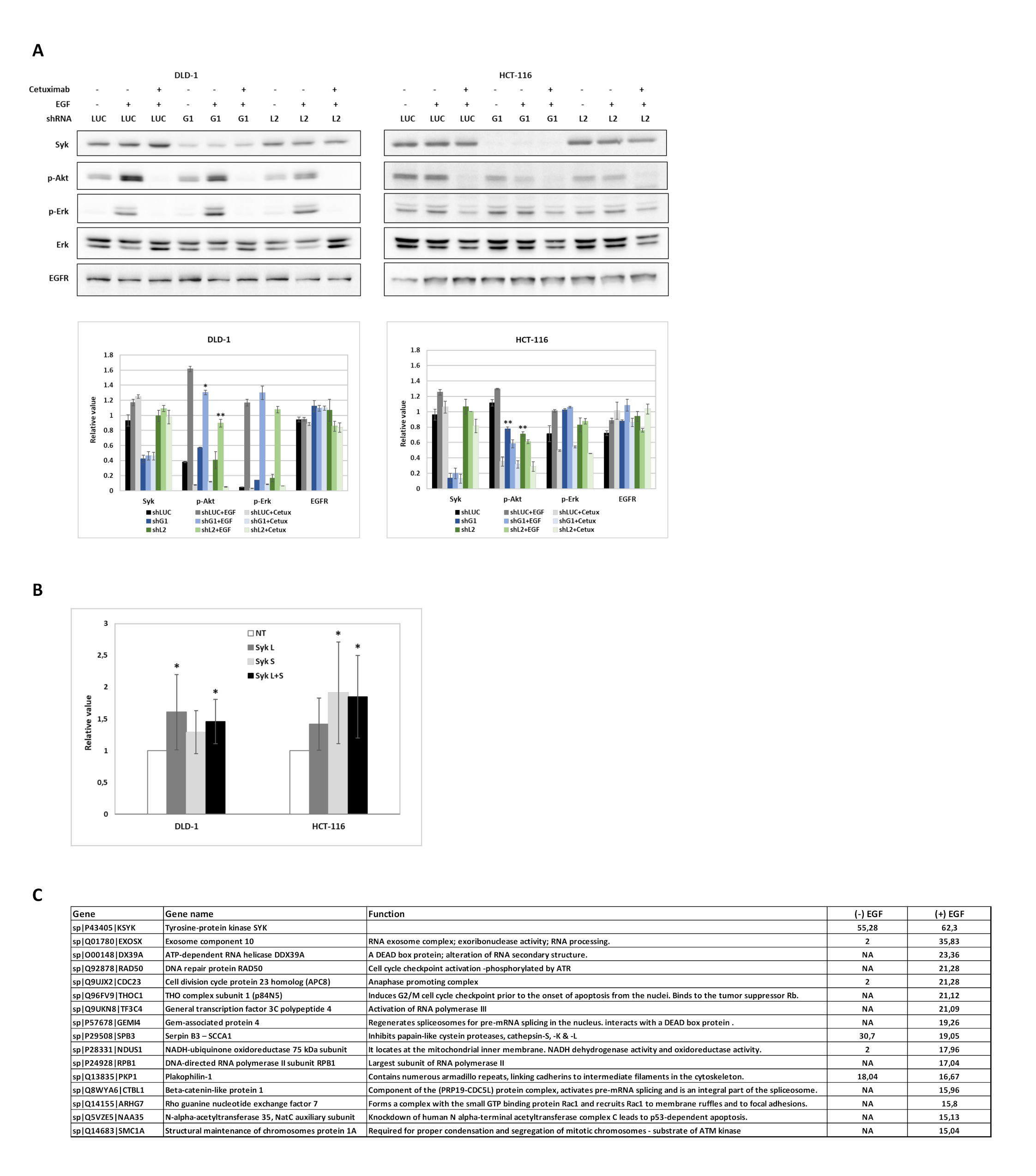

Supplement: S1 Fig — (A) The implication of Syk isoforms in CRC survival. Cells transduced with Syk shG1, Syk shL2 and shLUC (control) shRNAs were serum-starved, then treated with the recombinant human EGF (50 ng/mL) or a combination of EGF (50 ng/mL) + Cetuximab (50 μg/mL) for 10 minutes. Top panel- Protein extracts from cell lysates were analyzed by western blot using the indicated antibodies. Bottom panel- Densitometric quantification of immunoblot analyses of Syk, phospho-Akt, phospho-Erk and EGFR expression over control. (B) EGF promotes Syk alternative splicing by exon 9 inclusion / exclusion. HCT-116 and DLD-1 cell lines were serum starved for 16 h, then treated with EGF (50 ng/mL) for 15 minutes. The qPCR relative expression values for the overall gene expression Syk (L+S), long splice isoform Syk (L) and short splice isoform Syk (S) were normalized against the qPCR values obtained for untreated cells used as control (NT). The bar graph shows the changes in Syk splicing upon short-term EGF treatment. Error bars represent the mean ± SD of three independent experiments (*, P<0.05; **, P<0.01). (C) Identification of Syk interactome in serum starved and EGF-treated DLD-1 cells, by Mass spectrometry. Protein lysates from serum-starved and EGF-treated DLD-1 cells were incubated with agarose-conjugated anti-Syk 4D10 monoclonal antibody and agarose-conjugated mouse IgG (control) for 2 hours at 4°C. Beads were washed in lysis buffer, then they were resuspended in 1x Laemmli sample buffer and the protein contents were analyzed by SDS-PAGE. The table represents protein contents of Syk pulldowns in the absence of EGF (-EGF) and in the presence of EGF (+EGF) that scored over 15 and that were absent in the control IgG pulldowns. For data analysis, peptide and protein identifications were performed in Uniprot/Swiss- Prot2016_01 database by ProteinPilotTMSoftware V 4.5 (Sciex). (TIF) [file pone.0274390.s002.tif]

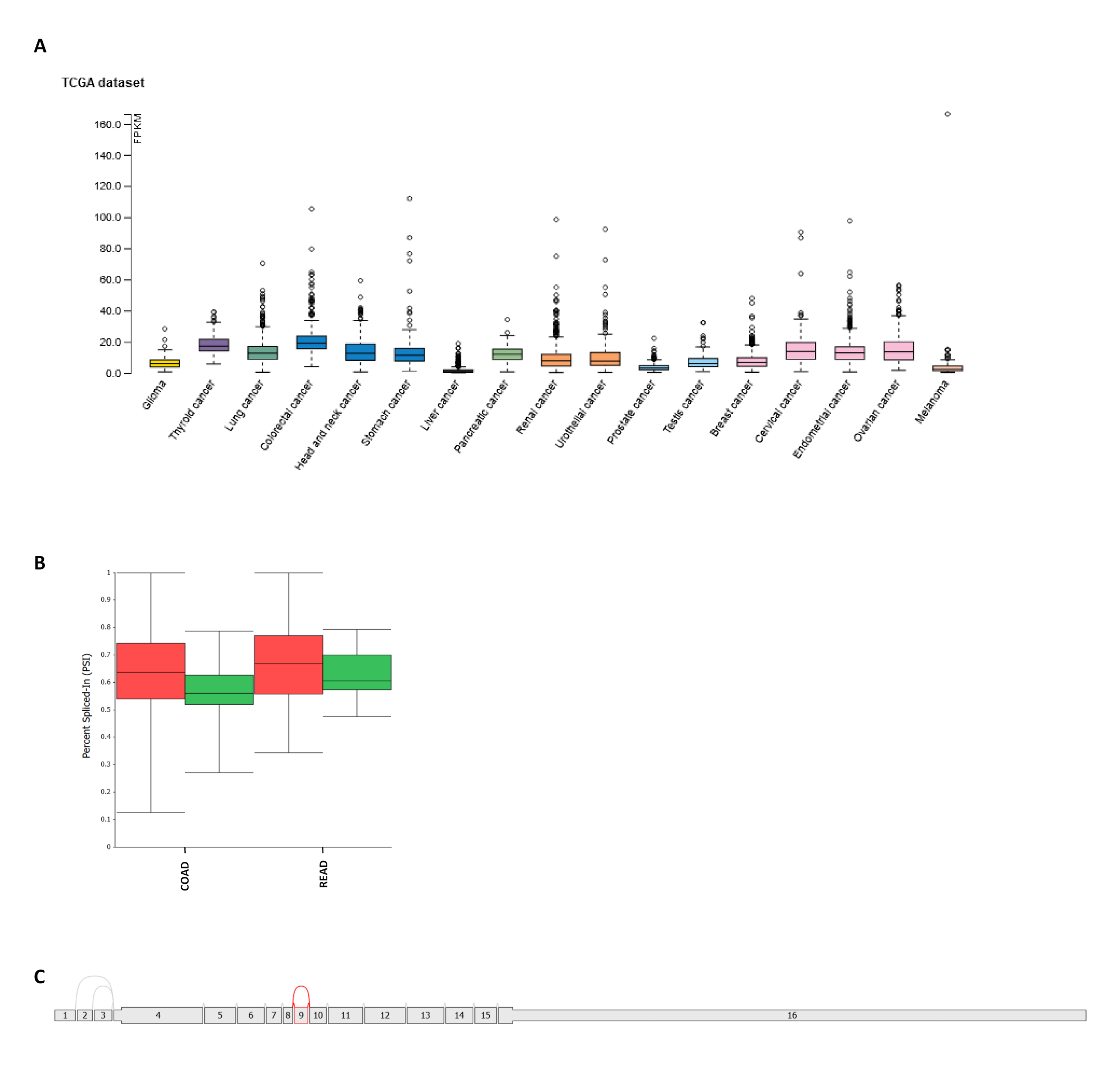

Supplement: S2 Fig — (A) Syk mRNA expression score in CRC tumors of The Cancer Genome Atlas (TCGA) (https://www.proteinatlas.org/ENSG00000165025-SYK/summary/rna). (B) Syk splicing variants expression in COAD and READ cohorts of the TCGA SpliceSeq database. The PSI (percent-splice-in) values correspond to the ratio of Syk (L) isoform expression over the total Syk transcripts calculated for the tumor tissues (red) and normal adjacent tissues (green) of the same patient [25]. (C) Genomic map of human Syk locus on chromosome 9 q22-2. (TIF) [file pone.0274390.s003.tif]

Figure 1B

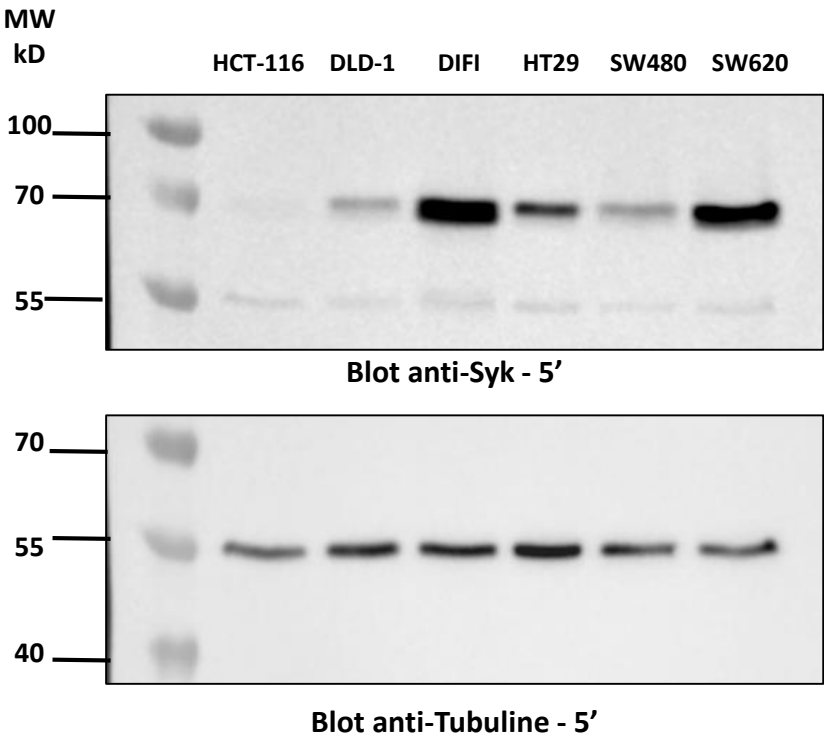

Figure 1E

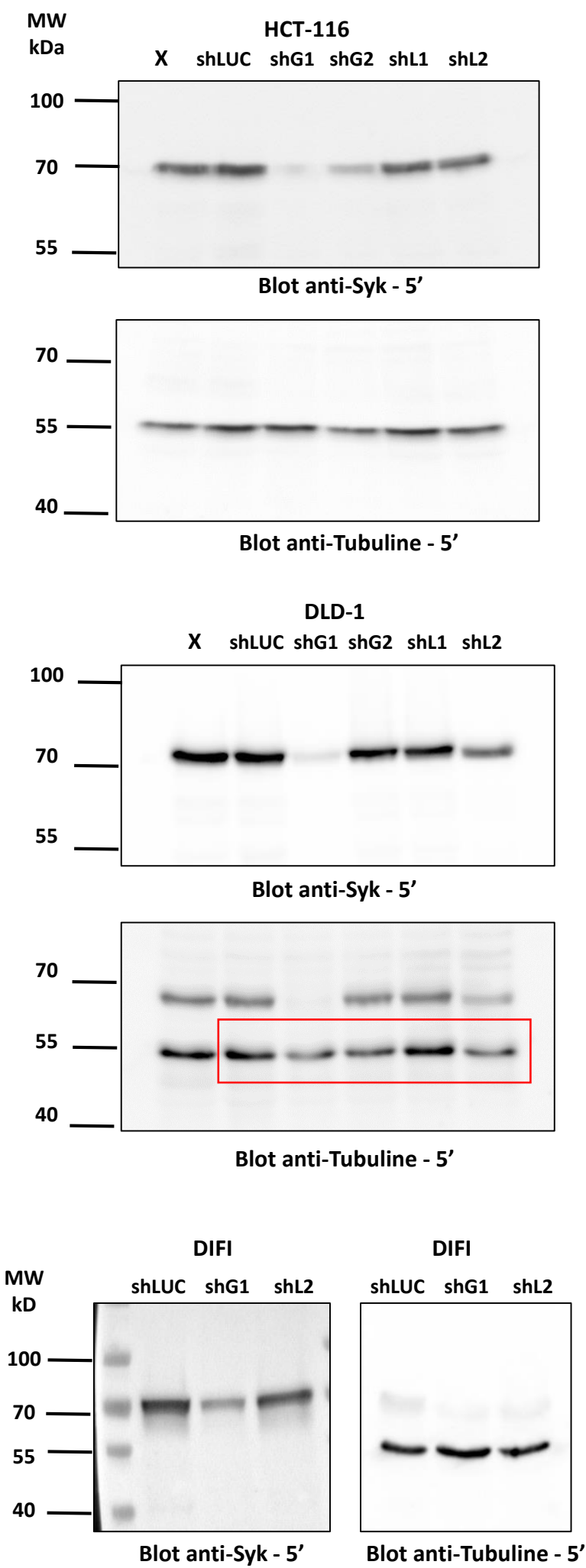

Figure 1D

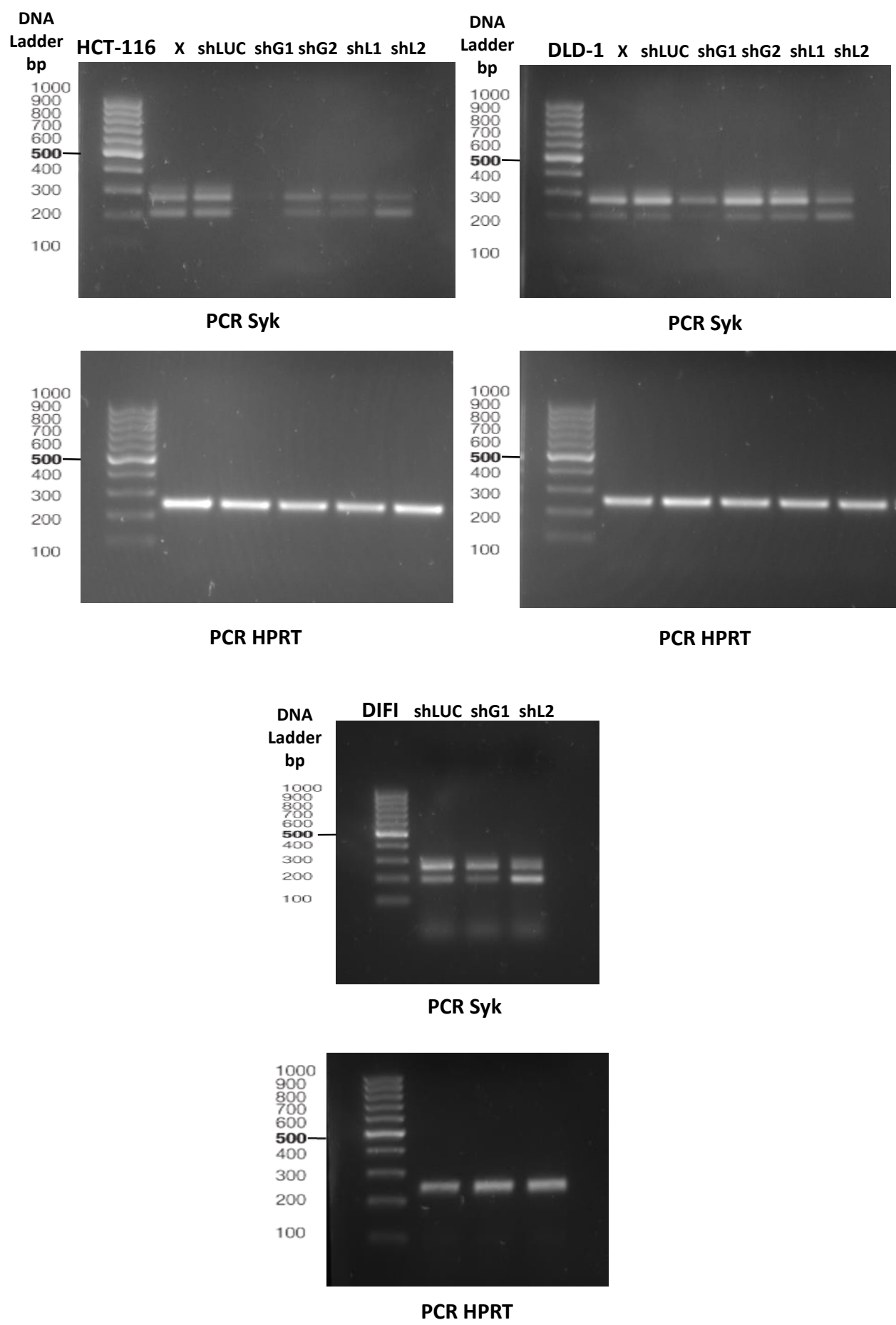

Figure 5A

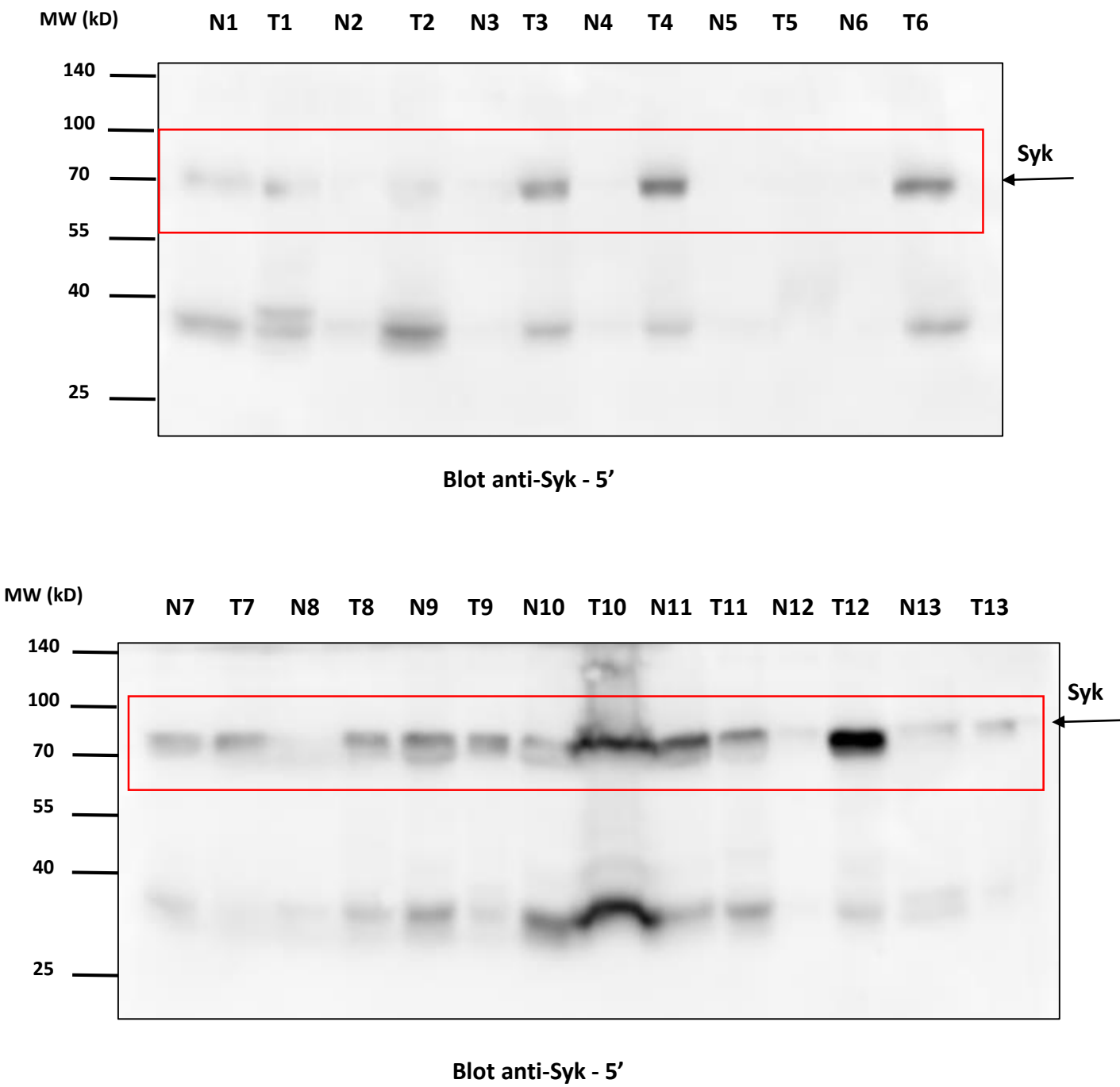

Figure 6F

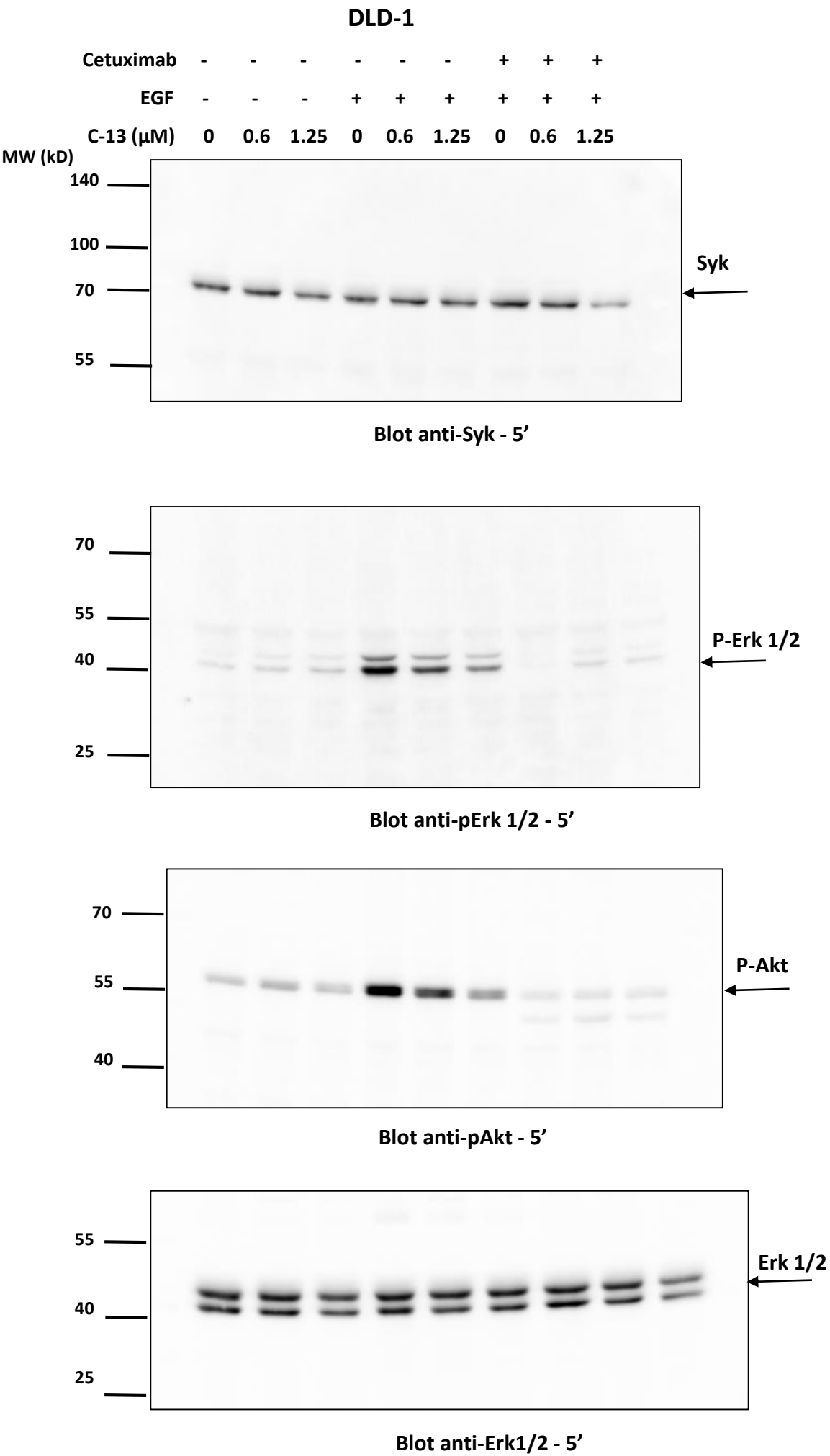

Supplementary Figure 1

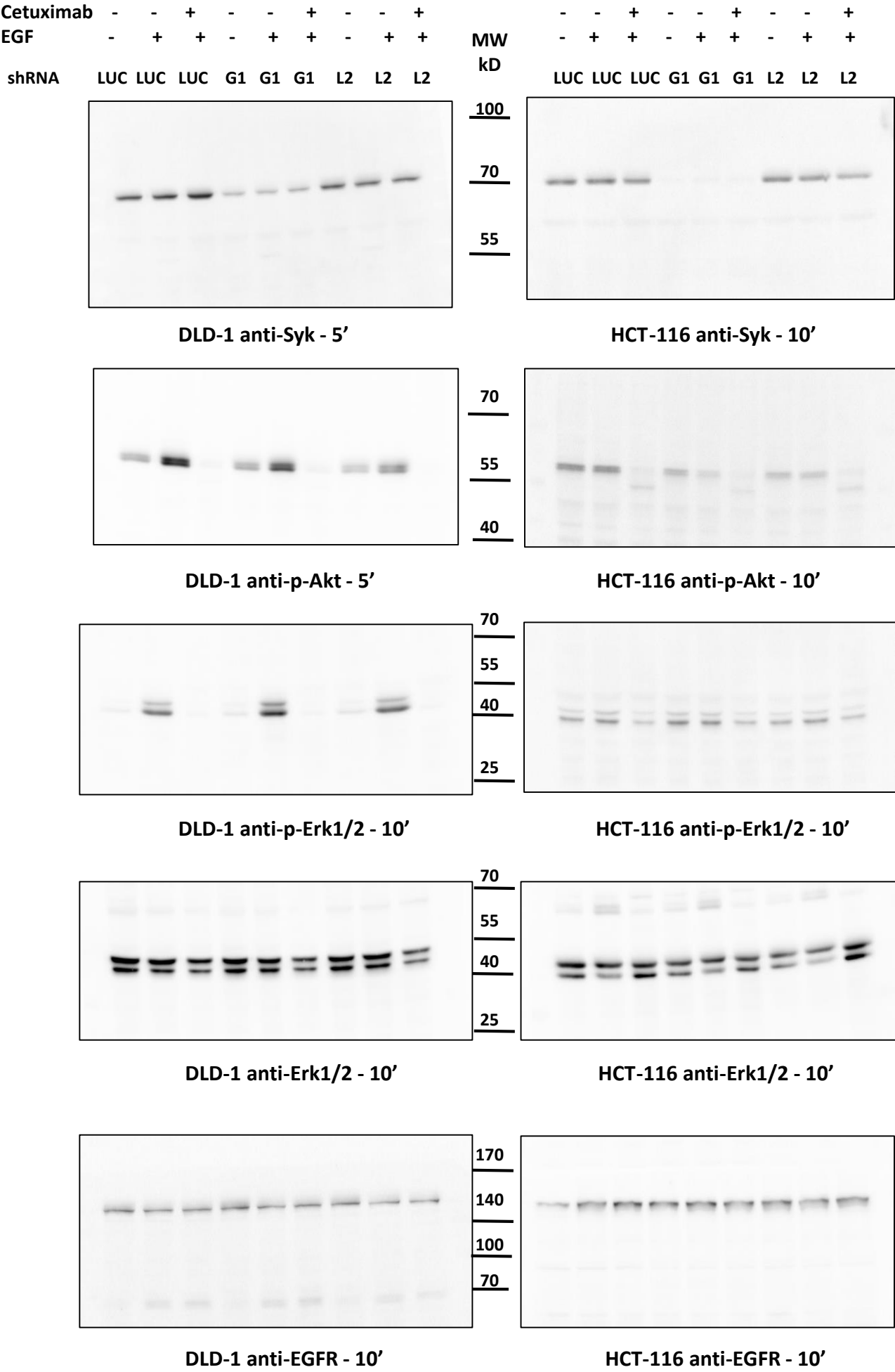

Supplement: S1 Raw images — (PDF) [file pone.0274390.s004.pdf]
